# Temporal Transcript Profiling Identifies a Role for Unfolded Protein Stress in Human Gut Ischemia-Reperfusion Injury

**DOI:** 10.1016/j.jcmgh.2021.11.001

**Published:** 2021-11-11

**Authors:** Anna M. Kip, Joep Grootjans, Marco Manca, M’hamed Hadfoune, Bas Boonen, Joep P.M. Derikx, Erik A.L. Biessen, Steven W.M. Olde Damink, Cornelis H.C. Dejong, Wim A. Buurman, Kaatje Lenaerts

**Affiliations:** 1 Department of Surgery, NUTRIM School of Nutrition and Translational Research in Metabolism, Maastricht University, Maastricht, the Netherlands; 2 Department of Pathology, CARIM School for Cardiovascular Diseases, Maastricht University Medical Center+, Maastricht, the Netherlands; 3 Department of Pediatric Surgery, Emma Children’s Hospital, Amsterdam University Medical Center, University of Amsterdam, Vrije Universiteit Amsterdam, Amsterdam, the Netherlands; 4 Institute for Molecular Cardiovascular Research, Rheinisch-Westfälische Technische Hochschule (RWTH) Aachen University Hospital, Aachen, Germany; 5 Department of General, Visceral, and Transplantation Surgery, RWTH Aachen University Hospital, Aachen, Germany

**Keywords:** Transcriptomics, Intestinal Ischemia-Reperfusion, Unfolded Protein Response, Human Intestinal Organoids, ATF4, activating transcription factor 4, BiP, binding immunoglobulin protein, C, control, CHOP, CCAAT/enhancer-binding protein homologous protein, eIF2α, eukaryotic translation initiation factor 2A, EM, Electron microscopy, ER, endoplasmic reticulum, GADD34, growth arrest and DNA-damage-inducible protein, GO, gene ontology, HIF1A, hypoxia-inducible factor 1-α, I, ischemia, IRE1, inositol-requiring enzyme 1, ISRIB, integrated stress response inhibitor, JNK, c-Jun N-terminal kinase, KEGG, Kyoto Encyclopedia of Genes and Genomes, MAPK, mitogen-activated protein kinase, mEGF, mouse epidermal growth factor, NF-κB, nuclear factor-κB, PERK, protein kinase R-like ER kinase, qPCR, quantitative polymerase chain reaction, R, reperfusion, ROCK, Rho kinase, UPR, unfolded protein response, XBP1, X-box binding protein 1, XBP1s, spliced X-box binding protein 1

## Abstract

**Background & Aims:**

Intestinal ischemia-reperfusion injury is a serious and life-threatening condition. A better understanding of molecular mechanisms related to intestinal ischemia-reperfusion injury in human beings is imperative to find therapeutic targets and improve patient outcome.

**Methods:**

First, the in vivo dynamic modulation of mucosal gene expression of the ischemia-reperfusion–injured human small intestine was studied. Based on functional enrichment analysis of the changing transcriptome, one of the predominantly regulated pathways was selected for further investigation in an in vitro human intestinal organoid model.

**Results:**

Ischemia-reperfusion massively changed the transcriptional landscape of the human small intestine. Functional enrichment analysis based on gene ontology and pathways pointed to the response to unfolded protein as a predominantly regulated process. In addition, regulatory network analysis identified hypoxia-inducing factor 1A as one of the key mediators of ischemia-reperfusion–induced changes, including the unfolded protein response (UPR). Differential expression of genes involved in the UPR was confirmed using quantitative polymerase chain reaction analysis. Electron microscopy showed signs of endoplasmic reticulum stress. Collectively, these findings point to a critical role for unfolded protein stress in intestinal ischemia-reperfusion injury in human beings. In a human intestinal organoid model exposed to hypoxia-reoxygenation, attenuation of UPR activation with integrated stress response inhibitor strongly reduced pro-apoptotic activating transcription factor 4 (ATF4)-CCAAT/enhancer-binding protein homologous protein (CHOP) signaling.

**Conclusions:**

Transcriptome analysis showed a crucial role for unfolded protein stress in the response to ischemia-reperfusion in human small intestine. UPR inhibition during hypoxia-reoxygenation in an intestinal organoid model suggests that downstream protein kinase R-like ER kinase (PERK) signaling may be a promising target to reduce intestinal ischemia-reperfusion injury. Microarray data are available in GEO (https://www.ncbi.nlm.nih.gov/gds, accession number GSE37013).

## Graphical abstract

**Figure:**
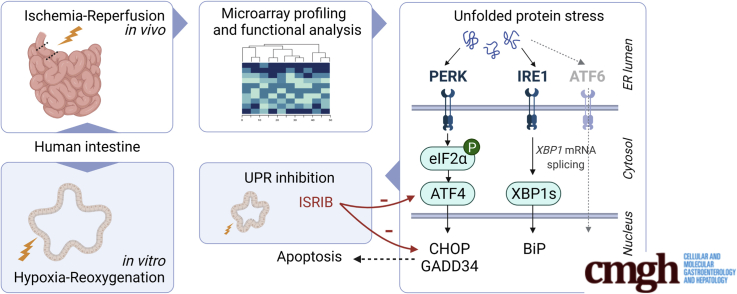

**Summary**

Ischemia-reperfusion of the human small intestine massively changed its transcriptional landscape. Functional analysis showed predominant regulation of the unfolded protein response. Pharmacologic inhibition of the unfolded protein response strongly reduced its pro-apoptotic signaling during hypoxia-reoxygenation in human small intestinal organoids.

The human intestine has an important role in nutrient and fluid uptake, while at the same time, it has to provide a protective barrier between the inner and outer milieu.^1^ For both the absorptive and barrier functions, the intestinal epithelium is dependent on sufficient oxygen supply.^2^ Under physiologic conditions, the intestinal mucosa experiences profound fluctuations in blood flow (eg, intestinal perfusion is enhanced after meal ingestion and is diminished considerably during physical exercise).^2^^,^^3^ In pathologic conditions, more severe impairment of intestinal perfusion may lead to intestinal ischemia, for instance, as a consequence of mesenteric thrombosis, shock, sepsis, vasculitis, or major surgery.^4^^,^^5^ In addition, it is well appreciated that epithelial hypoxia occurs secondary to inflammation, which plays a crucial role in the pathophysiology of inflammatory bowel disease.^2^^,^^6^ During interruption of blood supply, metabolic disturbances with mitochondrial dysfunction and energy deficiency damage the enterocytes. Reperfusion of the ischemic tissue triggers an inflammatory response, with chemotactic recruitment of neutrophils, promoting an even more hypoxic environment,^7^ and further destruction of the intestinal mucosa, leading to barrier compromise.^8^

Given the importance of ischemia-reperfusion injury as a pathophysiological phenomenon in a variety of diseases, it is imperative to understand the interactions between metabolic changes caused by ischemia-reperfusion, and the molecular mechanisms related to intestinal epithelial dysfunction and inflammation. Our knowledge on these interactions is derived mostly from studying animal models,^9^ and although our knowledge on intestinal ischemia-reperfusion injury in human beings has improved in the past decade,^10^ the key players in its pathophysiology remain obscure. In this study, we set out to investigate the molecular mechanisms driving intestinal ischemia-reperfusion–induced pathology, using a human experimental model.^8^ The experimental framework enabled consecutive sampling of intestinal tissue specimens to monitor the temporal behavior of biological processes over a period of progressive injury and subsequent initiation of repair, through analysis of their transcriptional profiles.

This led us to decipher the coordinated gene regulation events upon ischemia-reperfusion stress in the human intestine. This knowledge provides a basis for the development of targeted interventions to diminish intestinal ischemia-reperfusion–induced mucosal injury and inflammation and restore intestinal homeostasis. Based on our analysis, we selected one of the predominantly regulated processes and further investigated its potential as a therapeutic target in ischemia-reperfusion injury, using a human intestinal organoid model.^11^

## Results

### The Progressively Changing Transcriptional Landscape of the Ischemically Injured Human Intestine

**Figure 1:**
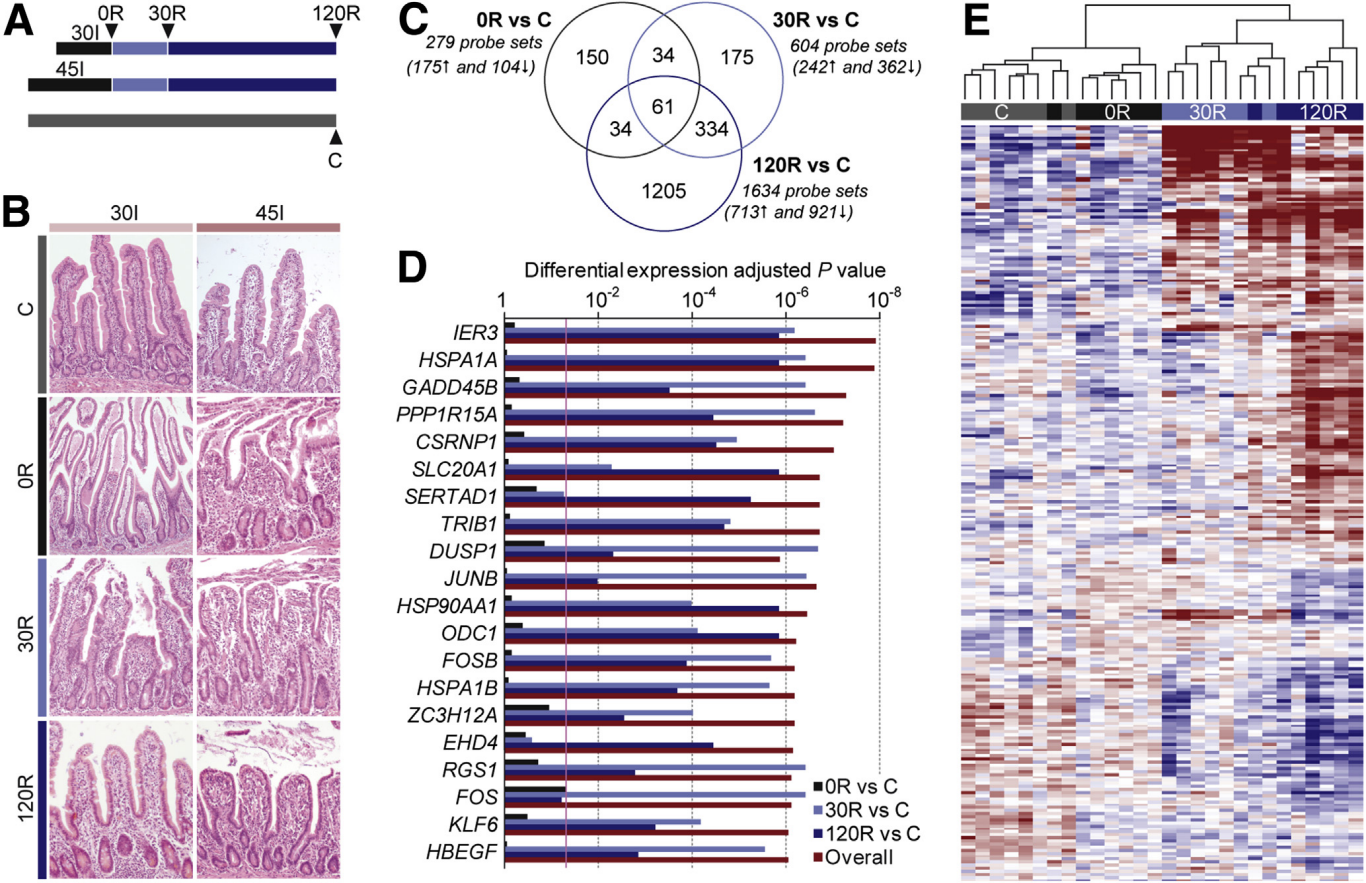
**Transcriptional changes in the human intestine in response to ischemia-reperfusion.** (*A*) Schematic overview of experimental protocol followed to harvest intestinal samples exposed to 30I or 45I of normothermic ischemia without reperfusion (0R, *blackbars*), and with 30R (*light blue bars*) and 120R (*dark blue bars*). Control specimens (C) not exposed to ischemia-reperfusion stress also were collected from all patients (*light grey bar*). (*B*) Representative images of H&E staining of intestinal tissue harvested according to the scheme shown in panel *A*. (*C*) Venn diagram of Boolean relationships between sets of differentially expressed genes in response to intestinal ischemia-reperfusion (N = 7 patients). The total number of regulated genes is shown per comparison (with the number of up-regulated and down-regulated genes shown in parentheses). (*D*) Top 20 differentially expressed genes in the human intestine in response to ischemia-reperfusion. (*E*) The dendrogram shows hierarchical clustering based on the top 500 significantly changed probe sets in response to ischemia-reperfusion injury in 7 patients. Probe sets for which the abundance was above the mean are shown in red, below the mean are shown in blue, and equivalent to the mean are shown in white.

To investigate the effect of disturbed blood supply on the human small intestine, we first performed a histologic assessment of ischemia-reperfusion–exposed tissue harvested according to the protocol shown in Figure 1*A*. The extent of mucosal injury increased progressively with ischemia duration, that is, 30 minutes of ischemia (30I) and 45I (Figure 1*B*). Most severe tissue damage was apparent after 30 minutes of reperfusion (30R), especially at the villi tips. Within 120R, damage was almost completely restored in the 30I group, whereas in the 45I group the epithelial lining still was interrupted.

To understand the molecular mechanisms underpinning the human intestinal response to ischemia-reperfusion, a microarray study was performed. Genome-wide expression profiles of consecutive specimens harvested during human intestinal ischemia-reperfusion were generated from 7 patients (the 30I and 45I groups), (Supplementary Table 1 patient characteristics). Three tissue specimens harvested during ischemia-reperfusion at 0R, 30R, and 120R, as well as a normally perfused control sample (C), were profiled for each individual. Overall, the signal intensity of 1993 probe sets (representing 1847 unique genes) changed significantly (adjusted *P* ≤ .05) in response to ischemia-reperfusion. The number of differentially expressed transcripts increased steadily with reperfusion time (Figure 1*C*), which represents the highly dynamic nature of the response. Figure 1*D* shows the top 20 differentially expressed genes in the human intestine in response to ischemia-reperfusion. The 50 most up-regulated and down-regulated genes per comparison are shown in Supplementary Table 2. Hierarchical clustering based on the top 500 differentially expressed genes showed distinct clustering of the majority of control samples and samples exposed to different conditions of ischemia and reperfusion (Figure 1*E*). Moreover, 2 major clusters strictly divided the control and ischemic tissue from the tissue exposed to reperfusion, indicating that the most dramatic transcriptional changes occurred when perfusion was restored after an ischemic insult.

### Fundamental Biological Processes and Pathways Activated During Human Intestinal Ischemia-Reperfusion

**Figure 2:**
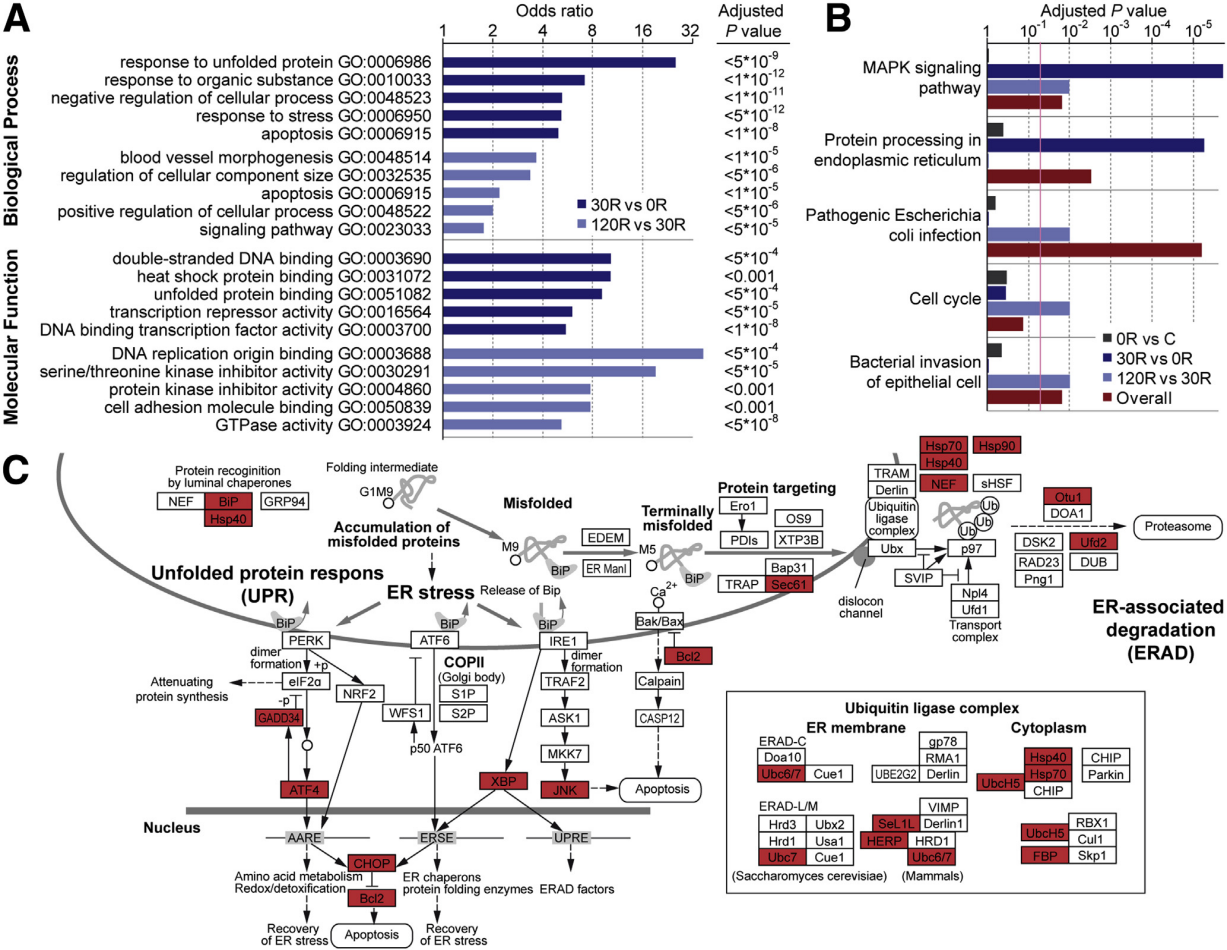
**Mapping transcriptional changes to biological functions.** (*A*) Top 5 GO terms identified by GO-based enrichment analysis in the category of Biological Process and Molecular Function for significantly differentially expressed genes during reperfusion. The x-axis (log-scale) indicates the odds ratio that a GO term is enriched in the selected category. Several GO terms were excluded from the list because of overlap based on similar groups of genes. See Supplementary Table 3 for the top 30 GO terms for biological processes (including overlapping terms) and all GO terms for molecular function. (*B*) Top 5 significantly over-represented KEGG pathways in the human intestine in response to ischemia-reperfusion. All significantly over-represented pathways (adjusted *P* < .05) can be found in Supplementary Table 4. (*C*) Top perturbed pathway during reperfusion (30R) of ischemically injured intestine, namely Protein processing in ER (modified from KEGG pathway hsa04141). Significantly differentially expressed genes are indicated in red. Differential expression is evident in the regions of ER stress and ER-associated degradation (ERAD), which is a process responsible for ubiquitination and degradation of terminally misfolded proteins through the proteasome. GTPase, guanosine triphosphatase.

To facilitate functional interpretation of genes influenced by ischemia-reperfusion in the human intestine, we first performed gene ontology (GO) term analysis in the categories of biological process and molecular function (Figure 2*A*). In the first 30 minutes of reperfusion, functional categories were associated primarily with failure to properly fold and dispose of damaged proteins. The GO terms “response to unfolded protein,” “response to stress,” “heat shock protein binding,” and “unfolded protein binding” were enriched significantly. Prolonged reperfusion was characterized by the GO terms “blood vessel morphogenesis,” “apoptosis,” and “cell adhesion molecule binding,” which all are indicative of intestinal remodeling. In Supplementary Table 3, the list of the 30 most significantly enriched GO terms is shown for biological processes, and all GO terms with respect to molecular function. Functional enrichment analysis based on pathways in the Kyoto Encyclopedia of Genes and Genomes (KEGG) database showed the most significant over-representation of genes involved in the mitogen-activated protein kinase (MAPK) signaling pathway and protein processing in endoplasmic reticulum (ER) within the 0R to 30R time frame (Figure 2*B*, Supplementary Table 4). The following time frame, that is, between 30R and 120R, was characterized by signs of bacterial invasion and cell proliferation with over-representation of the pathways of pathogenic *Escherichia coli* infection, cell cycle, and bacterial invasion of epithelial cells.

In general, functional GO and KEGG pathway analyses strongly imply a response of the intestinal tissue toward unfolded protein in the ER during early reperfusion. Differentially expressed genes in the pathway protein processing in the ER are shown in Figure 2*C*. Co-activation of the ER stress pathway and MAPK signaling pathway, with altered gene expression particularly in the c-Jun N-terminal kinase (JNK) and p38 MAPK cascade, during early reperfusion, provides a plausible link between ER stress on the one hand, and inflammation and apoptosis on the other hand. Indeed, ER-stress signaling molecules are known to trigger the inflammatory signaling components JNK and nuclear factor-κB (NF-κB).^12^ Both factors and their downstream targets (eg, c-Jun, c-Fos, NF-κB inhibitor alpha, and Tumor necrosis factor alpha-induced protein 3) were found to be induced in the human small intestine exposed to ischemia-reperfusion.

### Dynamic Gene Regulatory Network Analysis Shows a Key Role for Hypoxia-Inducible Factor 1-α in Regulating the Unfolded Protein Response During Ischemia-Reperfusion

**Figure 3:**
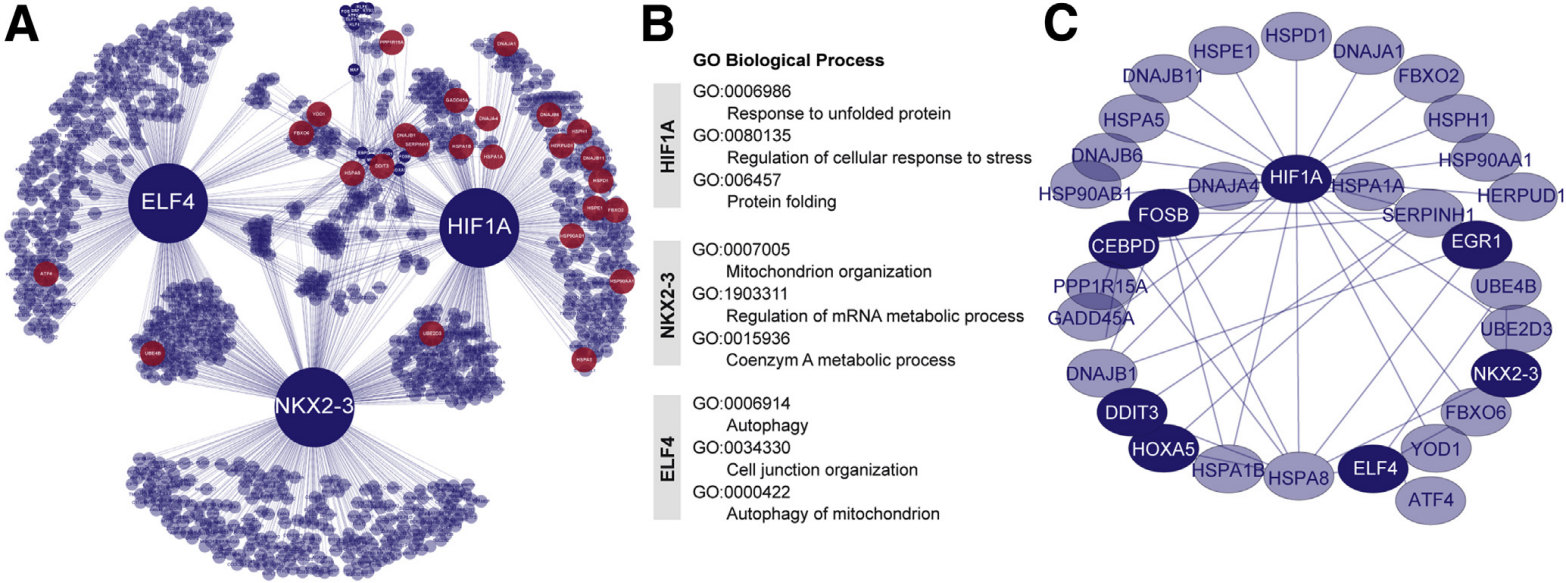
**Dynamic network of regulatory interactions in the ischemia-reperfusion–exposed human intestine shows a key role of HIF1A in the regulation of the response to unfolded protein.** (*A*) Network of regulatory interactions involved in the response to ischemia and subsequent reperfusion in the human intestine. The nodes represent the regulators and their targets, and the regulatory interactions are the edges. All transcriptional regulators in the network are shown in dark blue. See Supplementary Table 5 for a list of transcription factors and the number of targets in the network. The 3 major transcriptional regulators in the network are HIF1A, NKX2-3, and ELF4. Genes annotated to the GO term response to unfolded protein are marked in red. (*B*) The top over-represented biological processes are shown for HIF1A, NKX2-3, and ELF4 predicted targets in the network. Functional enrichment analysis for GO biological processes was performed using METASCAPE. The complete lists of enriched biological processes are shown in Supplementary Table 6. (*C*) Genes in the category response to unfolded protein in relation to HIF1A and the transcriptional regulators (dark blue). mRNA, messenger RNA.

Next, to further delineate molecular targets that primarily dictate the response to ischemia-reperfusion in the human intestine, we identified core networks essential for the adaptation of damaged intestinal tissue at the transcriptional level. Regulatory relationships among the differentially expressed genes were calculated based on their temporal variation in transcript abundance in response to ischemia-reperfusion. We selected known transcription factors as network hubs. Of 96 differentially expressed transcription factors in our set, 18 appeared in the network. Three transcription factors, hypoxia-inducible factor 1-α (encoded by *HIF1A*), homeobox protein Nkx-2.3 (encoded by *NKX2-3*), and ETS-related transcription factor Elf-4 (encoded by *ELF4*), were identified as predominant regulators in our model (Supplementary Table 5). The assembly of regulatory interactions is shown in Figure 3A. Remarkably, functional analysis of HIF1A targets in the category of biological process showed highly significant enrichment of the GO terms response to unfolded protein (*P* < 2.6∗10^-15^), regulation of cellular response to stress (*P* < 2.1∗10^-13^), and protein folding (*P* < 3.0∗10^-13^) (Figure 3*B*, Supplementary Table 6). These processes did not appear in the list of enriched GO terms for NKX2-3 and ELF4 targets (Figure 3*B*, Supplementary Table 6). All genes annotated to the category response to unfolded protein were marked red in the network and, interestingly, the majority was centered around HIF1A (Figure 3*A*). The genes involved and their relationship to HIF1A and the other linked network hubs are shown in Figure 3*C*. These data imply a key role for HIF1A in the response of the human intestine toward unfolded protein accumulation during ischemia-reperfusion.

### Ischemia-Reperfusion Triggers ER Stress in the Human Intestinal Epithelium

**Figure 4:**
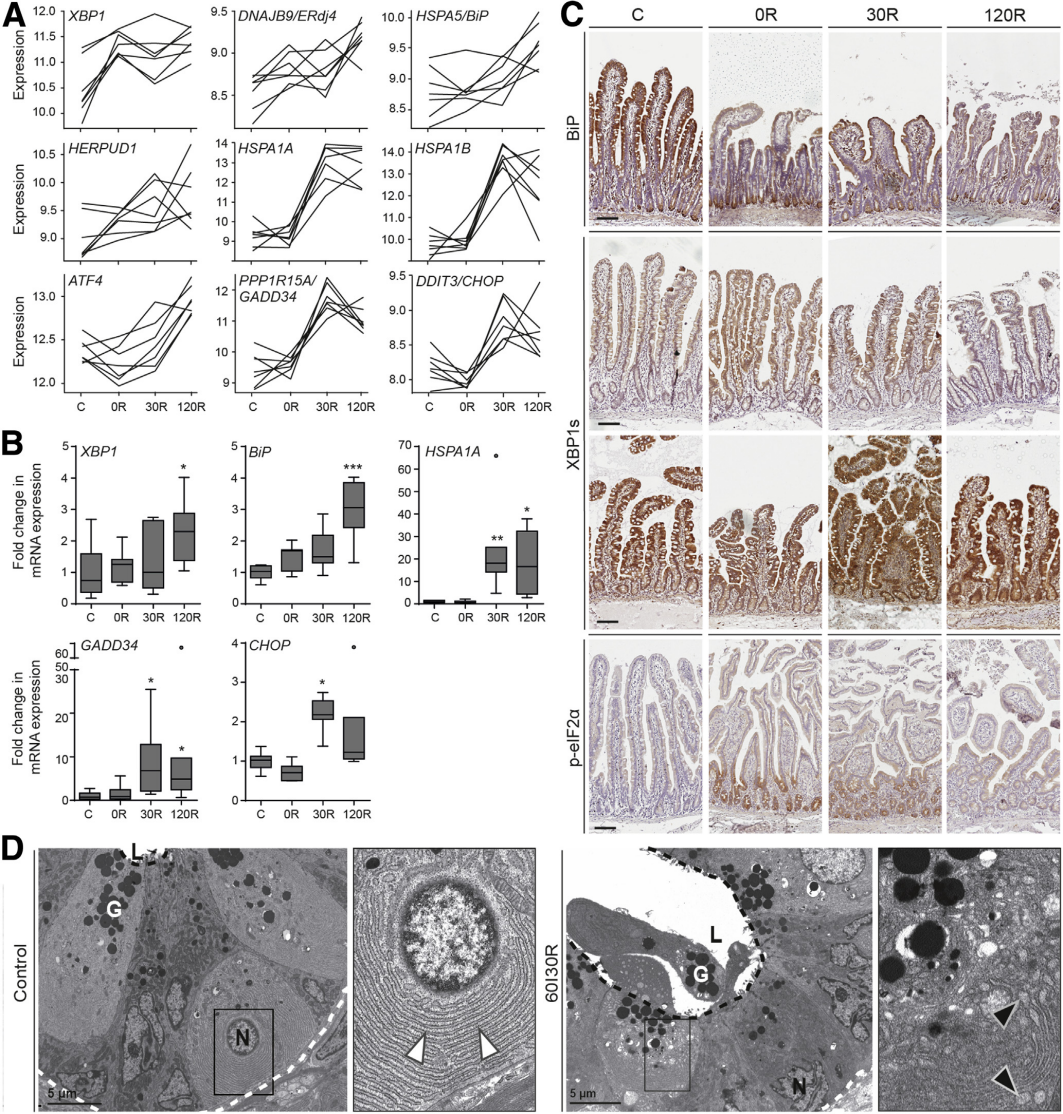
**Signs of ER stress in the human small intestine exposed to ischemia-reperfusion.** (*A*) A selection of genes involved in the response to unfolded protein is up-regulated markedly in the intestine exposed to ischemia-reperfusion. The y-axis indicates the log_2_ normalized gene expression. The data show the temporal nature of gene expression and show the high degree of homogeneity between patients (N = 7). *Upper panel* and *middle panel*: Genes involved in the IRE1 arm of the UPR; *lower panel*: PERK signaling. (*B*) qPCR analysis of *XBP1*, *BiP*, *HSPA1A*, *GADD34*, and *CHOP* (N = 7). Data are presented as fold change compared with C, and shown in Tukey boxplot (*dots* indicate outliers). ∗*P* < .05 vs C, ∗∗*P* < .01 vs C, and ∗∗∗*P* < .001 vs C. (*C*) Immunohistochemical staining of BiP, XBP1s, and phospho- eIF2α (p-eIF2α). Representative images from 1 or 2 patients are shown. *Scale bar*: 100 μm. (*D*) Electron microscopy images of control (*upper panel*) and ischemia-reperfusion–exposed (60I30R, *lower panel*) jejunal crypt base epithelium. Higher-magnification views of the *boxed regions* are shown on the *right*. Paneth cells, characterized by cytoplasmic granules (G), are presented because of their extensive ER. Control tissue shows normally structured ER (*white arrowheads*). Ischemia-reperfusion-exposed tissue shows vacuoles representing dilated ER lumina (*black arrowheads*). A *white dashed line* outlines the outer margin of the crypts; a *black dashed line* demarcates the luminal side. N, nucleus; L, lumen. mRNA, messenger RNA.

As shown earlier, extensive analysis of transcriptional profiles at the level of pathways, functions, and regulatory networks points to the relevance of protein folding stress in the ischemia-reperfusion–injured small intestine. Accumulation of unfolded proteins within the ER induces an adaptive stress response known as the unfolded protein response (UPR). Expression of X-box binding protein 1 (XBP1), a critical effector of the UPR, was up-regulated significantly after ischemia-reperfusion (Figure 4*A*). Upon UPR activation, inositol-requiring enzyme 1 (IRE1) cleaves 26 bp from XBP1 messenger RNA to yield spliced XBP1 (XBP1s), whose product transcriptionally activates major portions of the UPR.^13^ In a previous study, we reported increased levels of XBP1s during human intestinal ischemia-reperfusion.^14^ Here, expression of known XBP1s targets were up-regulated significantly and included binding-immunoglobulin protein (BiP, encoded by *HSPA5*), DNAJB9 (also known as ERdj4), and HERPUD1 (Figure 4*A*, upper and middle panels).^13^^,^^15^^,^^16^ Increased *XBP1* and *BiP* expression levels were confirmed by quantitative polymerase chain reaction (qPCR) and were particularly enhanced at 120R (Figure 4*B*). In addition, the localization of IRE1 signaling was examined by immunohistochemistry. In control tissues, BiP protein was expressed both in the villi and the crypt base, whereas after ischemia-reperfusion, BiP staining was evidently present in the crypt base (Figure 4*C*). Staining of XBP1s showed expression throughout the intestinal epithelium as well as in the lamina propria. Its abundance seemed to be increasing during ischemia-reperfusion in either the villus epithelium, or lamina propria, without showing a consistent picture among different patients (Figure 4*C*). Heat shock protein family A (Hsp70) member 1A/B (encoded by *HSPA1A/B* in Figure 4*A*) is known to enhance IRE1-XBP1 signaling upon binding to IRE1, and hence promotes adaptation to ER stress and cell survival.^17^ *HSPA1A/B* appeared in the top 20 list of differentially expressed genes (Figure 1*D*), and showed a huge induction of its expression at 30R and 120R, as validated by qPCR (Figure 4*B*).

Next to IRE1 activation, up-regulation of activating transcription factor 4 (ATF4), CCAAT/enhancer-binding protein homologous protein (CHOP, encoded by *DDIT3*), and growth arrest and DNA-damage-inducible protein (GADD34, encoded by *PPP1R15A*) (Figure 4*A*) are indicative of parallel activation of the protein kinase R-like ER kinase (PERK) pathway in response to human intestinal ischemia-reperfusion. The induction of PERK signaling during reperfusion also was confirmed by qPCR analysis (Figure 4*B*). Immunohistochemical staining of phosphorylated eukaryotic translation initiation factor 2A (eIF2α) showed that PERK activation was localized predominantly in the crypt region after ischemia (0R) and reperfusion (30R) (Figure 4*C*).

In addition to transcriptional activation of the UPR, we examined subcellular signs of ER stress using transmission electron microscopy. The intestine exposed to ischemia-reperfusion shows an enlarged ER with vacuoles in the Paneth cells, which is indicative of ER stress, whereas control tissue shows normally structured ER (Figure 4*D*).

### Inhibition of the UPR With Integrated Stress Response Inhibitor Decreases Pro-apoptotic UPR Signaling in a Human Intestinal Organoid Model

**Figure 5:**
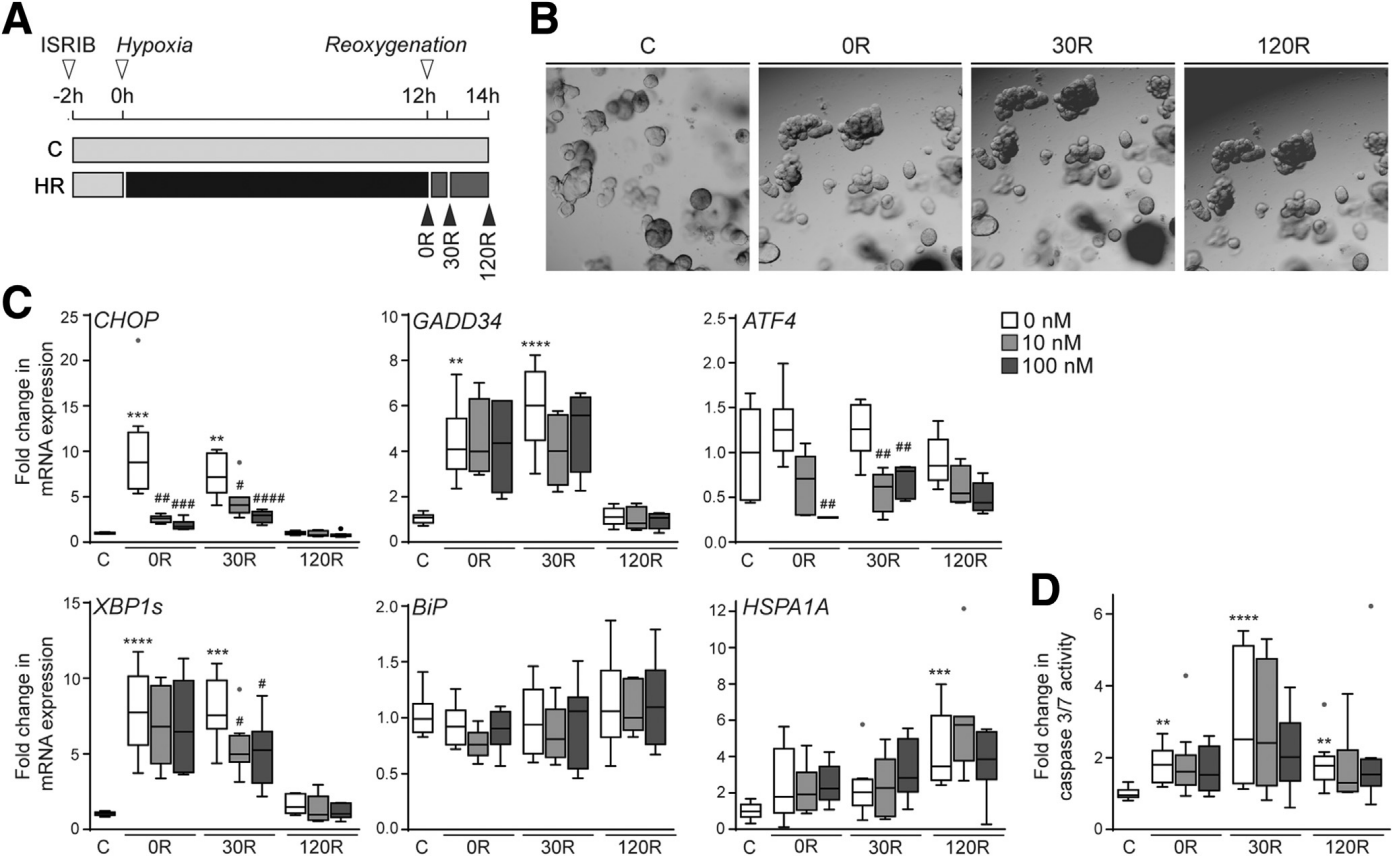
**Inhibition of the unfolded protein response decreases pro-apoptotic UPR signaling.** (*A*) Experimental set-up. Organoids were exposed to 12 hours of hypoxia without reoxygenation (0R), 30R, and 120R. ISRIB (0, 10, or 100 nmol/L) was added 2 hours before the start of hypoxia. Control samples were not exposed to hypoxia-reoxygenation (HR) (C). (*B*) Bright field images of human intestinal organoids during hypoxia-reoxygenation (images from the same culture are shown), and in control (magnification, 50×). (*C*) Messenger RNA (mRNA) expression of UPR-related genes *CHOP, GADD34, ATF4, XBP1s, BiP,* and *HSPA1A*. n = 3 per organoid line (N = 3). Data are presented as fold change compared with C, and shown in Tukey boxplot (*dots* indicate outliers). (*D*) Apoptosis measurement using a caspase 3/7 activity assay (Promega). Results are presented as fold change in luminescence compared with C (Tukey boxplot). n = 4 per organoid line (N = 3). Kruskal–Wallis with the Dunn multiple comparisons test was used to compare HR conditions with C (∗indicates significance), and per time point 10 and 100 nmol/L ISRIB were compared with no ISRIB (^#^indicates significance). ∗*P* < .05, ∗∗*P* < .01, ∗∗∗*P* < .001, and ∗∗∗∗*P* < .0001, ^#^*P* < .05, ^##^*P* < .01, ^###^*P* < .001, and ^####^*P* < .0001.

Our data point to an important role of ER stress and UPR signaling in the pathophysiology of ischemia-reperfusion injury of the human intestine. The UPR is key to cell survival, however, UPR signaling also can promote apoptotic cell death and aggravate injury if ER stress is sustained.^18^ To elucidate the functional role of the UPR in intestinal ischemia-reperfusion, we next investigated the effect of pharmacologic UPR inhibition in a human small intestinal organoid model. Organoids were exposed to hypoxia-reoxygenation as shown in Figure 5*A*, and treated with integrated stress response inhibitor (ISRIB), which reverses the effects of eIF2α phosphorylation.^19^^,^^20^ We hypothesized that inhibition of downstream PERK signaling with ISRIB is protective during hypoxia-reoxygenation by attenuating apoptosis.

No evident changes in organoid morphology were observed after hypoxia and reoxygenation, suggesting that organoids were not severely damaged (Figure 5*B*). A strong increase in gene expression of *CHOP* (7- to 10-fold), *GADD34* (4- to 6-fold), and *XBP1s* (8-fold) (Figure 5*C*) was observed in organoids in response to hypoxia-reoxygenation at 0R and 30R compared with C, indicating activation of both PERK and IRE1 branches of the UPR. In addition, in line with human ischemia-reperfusion data (Figure 4*B*), *HSPA1A* was increased significantly at 120R (Figure 5*C*). Treatment with small-molecule ISRIB resulted in significantly lower *ATF4* and *CHOP* expression at 0R and 30R, with *CHOP* levels close to control conditions. In contrast, ISRIB did not significantly change *GADD34* expression levels (Figure 5*C*). These data suggest pro-apoptotic ATF4-CHOP signaling was strongly reduced by ISRIB, whereas *GADD34* expression remained intact. In addition, *XBP1s* expression at 30R was lower with ISRIB exposure (Figure 5*C*), which shows the complex interaction between the different UPR branches.

To examine the effect of suppressed UPR signaling on epithelial cell death, caspase 3/7 activity was measured. A significant increase in apoptosis was observed in organoids at 0R, 30R, and 120R, with the highest apoptosis levels during early reperfusion (3-fold compared with C) (Figure 5*D*). Although there seemed to be a downward trend in apoptosis with increasing concentrations of ISRIB at 30R, no statistical significance was reached (Figure 5*D*).

## Discussion

Systems biology tools are invaluable in deciphering the complexity of the tissue response to injury, and in identifying novel targets for therapy. Through the transcriptomic profiling of consecutive stages of ischemia-reperfusion–exposed human intestinal tissue, and comprehensive analyses of transcriptome changes at the level of functions, pathways, and networks, we identified the key regulated biological processes activated during this tissue damage response.

Intriguingly, we found that, apart from expected regulated pathways involved in cell death, recovery, and inflammation, ischemia-reperfusion in the human intestine predominantly activates the cellular machinery to deal with unfolded protein stress. The sensitive protein-folding environment in the ER can be perturbed by various environmental factors, such as hypoxia, nutrient/energy depletion, oxidative stress, disturbances in ER Ca^2+^ balance, and increased secretory protein synthesis, which all occur during ischemia-reperfusion.^21^^,^^22^ This disturbance of ER homeostasis results in the accumulation of unfolded proteins, called ER stress, which initiates a series of prosurvival mechanisms, known as the UPR. The UPR alters the cellular transcriptional and translational program to cope with stressful conditions and resolve the protein-folding defect. However, if ER stress is sustained, UPR signaling can promote apoptotic cell death.^15^^,^^23^ Our data show crucial involvement of 2 principal UPR branches, namely IRE1 and PERK in UPR activation in the ischemia-reperfusion–exposed human intestine. The highly dynamic changes in these pathways likely are directed at restoring ER homeostasis by reducing the amount of new protein translocated into the ER lumen, by increasing degradation of ER-localized proteins, and by augmenting the ER folding capacity. Prosurvival UPR mechanisms include IRE1-induced splicing of XBP1 and PERK-induced phosphorylation of eIF2α (evidenced by more abundant expression in the crypt region at 0R and 30R) and after translational block. In contrast, the observed subsequent increase in ATF4, CHOP, and GADD34 expression, also downstream targets of PERK signaling, points to pro-apoptotic signaling after reperfusion.^24^ It should be mentioned that, in addition to the ER stress–induced UPR, mitochondrial stress also could be involved in UPR activation. More specifically, ATF4 and CHOP expression are induced by one of the branches of the mitochondrial UPR as well.^25^

Interestingly, network analysis of changed transcription factors and their targets has shown a key role for HIF1A in the intestinal response toward unfolded protein accumulation during ischemia-reperfusion. HIF1A is a transcription regulator that plays a crucial role in metabolic adaptation and cellular survival under hypoxic conditions.^26^^,^^27^ During ischemia, oxygen and nutrient insufficiency induces HIF1A signaling, which helps the intestine to adapt to these stressful conditions by inducing metabolic alterations, angiogenesis, and barrier protection.^28^^,^^29^ Activation of HIF1A has been reported in animal models of intestinal ischemia-reperfusion,^30^ and has been shown to be critical for cell survival in myocardial and renal ischemia-reperfusion injury.^27^^,^^31^^,^^32^ As HIF1A appeared to be a central transcriptional regulator of numerous genes involved in the response to protein folding stress, it can be postulated that part of its protective properties is attributable to activation of the protein-folding machinery. This is cohorent with previous studies on the crosstalk between hypoxia and the UPR in cancer models (reviewed by Bartoszewska and Collawn^26^), from which it was concluded that the required reduction in the cell’s energy demand during hypoxia is achieved in part via UPR-mediated suppression of translation. In addition, a link between HIF transcriptional activity and activation of PERK, ATF6, and IRE1 pathways in human endothelial cells, independent of ER stress, has been reported.^33^ Molecular chaperone HSPA1A, one of the targets in the HIF1A-centered network of ischemia-reperfusion–damaged intestine, has been shown to be regulated by HIF1A previously,^34^ and may provide another link with UPR activation.^17^ The biological relevance of the inferred network was shown by the presence of numerous known HIF1A target genes such as *VIM*, *ADAMTS1*, *PRNP*, and *CD55*.^35^, ^36^, ^37^, ^38^

Analysis of transcriptional profiles showed strong co-activation of the ER stress and MAPK signaling pathway. There is accumulating evidence on the extensive crosstalk between the ER stress response and the inflammatory response.^12^^,^^39^ In addition, ER stress and related UPR have been recognized to play a key role in intestinal pathology, such as inflammatory bowel disease.^40^^,^^41^ The UPR and inflammatory response are interconnected through various mechanisms, including reactive oxygen species production, calcium release from the ER, activation of inflammatory NF-κB, and MAPK signaling.^12^ Activation of NF-κB has been shown to be mediated via IRE1,^42^ PERK,^43^ and ATF6^44^ branches of the UPR. In addition, inflammatory response signaling via p38 and JNK MAPK pathways is known to be mediated by IRE1 and PERK signaling during ER stress.^45^, ^46^, ^47^ The co-activation of UPR signaling and inflammatory signaling during ischemia-reperfusion suggests that these processes cooperate during stress in the intestinal epithelium. Hence, besides exposure to luminal antigen as a consequence of the disrupted physical barrier,^8^ the ER stress response represents a plausible mechanism for exacerbation of inflammation in the ischemia-reperfusion–exposed human intestine.

In addition, ER stress also can be involved indirectly in inflammatory responses via its effects on Paneth cells, which are important players of the immunologic intestinal barrier.^48^ We previously reported Paneth cell apoptosis during reperfusion, which strongly correlated with the level of UPR activation.^14^ Because Paneth cells are invaluable for host defense against microbial invasion, loss of these cells can induce bacterial translocation, thereby provoking an inflammatory response. In line with this, the current study shows that during prolonged reperfusion, top regulated pathways were associated with signs of bacterial invasion, which is likely in part owing to loss of this essential innate immune barrier.

Altogether, our data point to a prominent role of UPR signaling in ischemia-reperfusion injury of the human intestine. Because UPR signaling is crucial in determining cell fate under ER stress, we explored the pharmacologic modulation of UPR signaling and its potential to protect against intestinal ischemia-reperfusion injury. General UPR inhibition with chemical chaperones has been reported to improve survival during ischemia-reperfusion in other organs. The fact that all arms of the UPR have prosurvival and prodeath potential makes it a challenging therapeutic target.^49^ More specific inhibitors of the UPR, such as ISRIB, have enhanced therapeutic potential compared with general inhibitors because they are less likely to cause adverse effects. Moreover, ISRIB is known to suppress the UPR effectively within a certain window of activation.^50^ The PERK-driven, ATF4-dependent induction of CHOP is considered to play a key role in UPR-related apoptotic cell death. We showed that inhibition of PERK signaling with its downstream inhibitor ISRIB reduced *ATF4* expression and almost completely prevented the huge induction in *CHOP* gene expression during hypoxia-reoxygenation in intestinal organoids. This indicates that ISRIB significantly reduced pro-apoptotic UPR signaling during hypoxia-reoxygenation. Because ISRIB acts downstream of PERK signaling by selectively reversing the effects of eIF2α phosphorylation, some of the protective effects of the UPR can be maintained.^19^^,^^20^^,^^51^ This is supported by a recent study reporting that ISRIB, but not a direct PERK kinase inhibitor, improved neuronal cell survival.^52^ Moreover, complete loss of PERK signaling has been shown to promote apoptosis.^53^

Surprisingly, ISRIB treatment also led to a moderate reduction in *XBP1s* messenger RNA after reoxygenation, indicating that modulation of PERK signaling also affects IRE1 activity. The importance of the coordination between these 2 branches of the UPR in cell fate decisions was reported recently by Chang et al,^54^ who showed that PERK signaling attenuates IRE1s protective activity during the terminal UPR apoptotic signaling, and, opposite to our findings, that ISRIB treatment enhanced IRE1 activation upon ER stress. This underlines the complexity of UPR dynamics, coordination between branches, and the effect of their modulation. The delicate balance between its protective role in mild ER stress, and its damaging effects in persisting or intense ER stress, is endorsed by studies showing the protective effect of ER stress preconditioning in kidney, liver, and heart ischemia-reperfusion-related pathologies.^55^, ^56^, ^57^, ^58^, ^59^ It would be very interesting to further explore the anticipated protective effect of mild pharmacologic ER stress induction before exposure to hypoxia-reoxygenation in intestinal organoids.

The heterogeneity of organoid cultures, as well as the fact that inhibition experiments were performed in human organoid lines derived from different individuals, likely accounts for the variation in functional outcome. Although the downward trend in apoptosis with increasing ISRIB concentration during reoxygenation did not reach statistical significance in the current experimental setting, these are promising results showing the potential of attenuating hypoxia-reoxygenation–induced cell death via a drastic decrease in pro-apoptotic ATF4/CHOP signaling. We believe that ISRIB has the potential to fine-tune the UPR in favor of survival, and to attenuate intestinal ischemia-reperfusion injury. However, thorough examination of the exact mechanisms and functional outcomes of ISRIB treatment during hypoxia-reoxygenation in vitro and ischemia-reperfusion in vivo is needed, as well as the effect on connected stress responses, such as MAPK, NF-κB, and HIF1A signaling.

In this study, we captured the dynamic modulation of genes in the human intestine subjected to ischemia-reperfusion. We provided a pathway-based framework of the intestinal damage response to ischemia-reperfusion in human beings, which may advance the understanding of numerous pathophysiological conditions with reduced intestinal perfusion and hypoxic signaling. We can conclude that ischemia-reperfusion massively changes the transcriptional landscape of the human gut, and, apart from the expected pathways involved in cell death, restoration, and inflammation, unfolded protein stress appeared to be of crucial importance among the regulated processes in human intestine exposed to ischemia-reperfusion. Therefore, we explored the potential of pharmacologic modulation of UPR signaling in the protection against intestinal ischemia-reperfusion injury. We showed that selective downstream PERK inhibition with ISRIB strongly reduced pro-apoptotic UPR signaling during hypoxia-reoxygenation in intestinal organoids. These findings suggest that downstream PERK targeting may be a promising strategy to treat ischemia-reperfusion–induced complications in the intestine, which is of utmost importance to reduce its high morbidity and mortality rates.

## Methods

### Ethics Statement

Human studies were approved by the Medical Ethics Committee (METC) of Maastricht University Medical Centre (METC 06-3-044, human ischemia-reperfusion model; METC 16-4-185, human intestinal organoid model) or Uniklinik Rheinisch-Westfälische Technische Hochschule Aachen (EK 206/09, human intestinal organoid model) and conducted according to the revised version of the Declaration of Helsinki (October 2008, Seoul). Written informed consent was obtained from all patients.

### Experimental Human Ischemia-reperfusion Model

The experimental protocol was performed as previously described.^8^ In short, 7 patients undergoing pancreaticoduodenectomy were included in this study. During pancreaticoduodenectomy, a variable length of jejunum routinely was resected in continuity with the head of the pancreas and duodenum as part of the surgical procedure. The terminal 6 cm of this jejunal segment was isolated and subjected to either 30 or 45 minutes of ischemia by placing 2 atraumatic vascular clamps across the mesentery. Meanwhile, surgery proceeded as planned. After ischemia, one third (2 cm) of the isolated ischemic jejunum was resected using a linear cutting stapler (0R). Next, clamps were removed to allow reperfusion, which was confirmed by regaining normal pink color and restoration of gut motility. Another segment of the isolated jejunum (2 cm) was resected similarly after 30R. The last part was resected after 120R. In addition, a 2-cm segment of jejunum, which was not subjected to ischemia-reperfusion but underwent similar surgical handling as the isolated part of the jejunum, was resected and served as internal control tissue (C). Figure 1*A* shows the experimental procedure that was followed to harvest tissue samples. Full-thickness tissue samples were immediately snap-frozen or formalin-fixed. Patients with obstructive jaundice underwent a stent procedure before surgery. All patients had normal bile flow at the time of surgery.

### Human Intestinal Organoid Model

Human tissue specimens of proximal jejunum, obtained from patients undergoing pancreaticoduodenectomy, were used for the generation of human small intestinal organoids.^11^ Organoids were embedded in basement membrane extract (Geltrex; Gibco, Carlsbad, CA) and cultured in growth medium containing Advanced Dulbecco’s modified Eagle medium/F12 (Gibco), 50 U/mL penicillin and 50 μg/mL streptomycin (Gibco), 1× Glutamax (Gibco), 10 mmol/L HEPES (Gibco), 1× B27 (Gibco), 1× N2 (Gibco), 50% vol/vol Wnt3a-conditioned medium, 20% vol/vol Rspondin-1–conditioned medium, 10% Noggin-conditioned medium, 50 ng/mL mouse epidermal growth factor (Gibco), 10 mmol/L nicotinamide (Sigma-Aldrich, St. Louis, MO), 1.25 mmol/L *N*-acetyl cystein (Sigma-Aldrich), 500 nmol/L A83-01 (transforming growth factor β inhibitor; Sigma-Aldrich), 10 mmol/L gastrin I (Sigma-Aldrich), and 10 μmol/L SB202190 (p38 MAPK inhibitor; Sigma-Aldrich). Rho kinase inhibitor Y-27632 (10 μmol/L; Abmole Bioscience, Houston, TX) was added to the medium when organoids were generated, after passaging and thawing.

To mimic ischemia-reperfusion, organoids were exposed to 12 hours of hypoxia (<1.0% O_2_, 5% CO_2_) and 2 hours of reoxygenation (21% O_2_, 5% CO_2_) as described previously.^11^ Organoids were harvested for analysis immediately after 12 hours of hypoxia without reoxygenation (0R), after 30R and 120R, and without hypoxic exposure (C). Experiments were performed in organoid lines derived from 3 patients. ISRIB (Sigma) was used as an inhibitor of the unfolded protein response. Organoids were exposed to hypoxia-reoxygenation with or without ISRIB, in concentrations of 10 nmol/L and 100 nmol/L. ISRIB was added to the medium 2 hours before hypoxia-reoxygenation.

### Microarray Analysis

#### RNA isolation

Total RNA was isolated from snap-frozen tissue samples (28 samples of 7 patients) using the AllPrep DNA/RNA/Protein kit (Qiagen, Hilden, Germany). In short, jejunal samples were crushed with a pestle and mortar in liquid nitrogen. Disruption and homogenization of the tissue was performed using an Ultra Turrax Homogenizer (IKA Labortechnik, Staufen, Germany) in lysis buffer containing β-mercaptoethanol (Promega, Madison, WI). RNeasy spin columns were used to bind RNA. Columns were washed and RNA was eluted in RNase-free water. RNA samples were treated with DNase (Promega). RNA quantity and quality were measured using the NanoDrop spectrophotometer (NanoDrop, Wilmington, DE) and the Agilent 2100 Bioanalyzer (Santa Clara, CA), respectively. All RNA samples included in the expression analysis had an RNA integrity number greater than 7.

#### Microarray analysis

Whole-genome expression analysis was performed using Illumina Human HT-12_V3_expression arrays (Illumina, San Diego, CA) on material collected from 7 patients (Supplementary Table 1). Four tissue specimens per patient were analyzed, namely intestinal tissue exposed to 30 or 45 minutes of ischemia (0R), with 30R and 120R, and a control sample (C). Per sample, 200 ng RNA was used for antisense RNA synthesis, amplification, purification, and labeling with the Illumina TotalPrep 96 RNA Amplification Kit (Applied Biosystems/Ambion, Austin, TX) according to the manufacturer's protocol. A total of 750 ng of complementary RNA was randomly hybridized to the Illumina arrays and scanned immediately on the Illumina BeadArray Reader. These microarrays contain 48,813 different probes targeting 37,812 different genes; some genes are targeted by more than 1 probe. The resulting data were quality-checked and extracted using Illumina GenomeStudio v 1.1.1 software without normalization and background subtraction. Raw BeadStudio (Illumina) output text files were uploaded in R (http://www.R-project.org) statistical environment by the Bioconductor^60^ software package lumi. Variance stabilizing transform and robust spline normalization were chosen as preprocessing methods; to minimize batch effects and provide an adequate statistical power, the whole data set was preprocessed simultaneously as a whole. A filter was applied to the detection calls, excluding all probes that were negative (with a 0.01 confidence) in 3 individuals or more, to minimize the technical noise in the analysis and to restrict the statistical adjustment for repeated tests to the informative data only. Microarray data are available in GEO (https://www.ncbi.nlm.nih.gov/gds, accession number: GSE37013).

#### Differential expression analysis

Time-course differential analysis was performed using Bioconductor package limma with time points as factors, and corrections for patient-IDs effect. Statistical significance was corrected for repeated testing using a false detection rate method. The threshold for acceptance was set to *P* ≤ .05. Illumina IDs were translated into Entrez ID by lumiHumanAll.db, which is assembled using data from public repositories. GO enrichment analysis of the differentially expressed genes was performed with GeneAnswers querying GO Biological Process, GO Molecular Function, and KEGG. Pathway enrichment analysis was performed with signaling pathway impact analysis, maintaining the standard scoring settings. All data were organized by patient IDs and time points using the R package longitudinal. Subsequently, Empirical Bayes Estimation of Dynamic Bayesian Networks was used to reverse engineer the expression regulation network linking the significantly differentially expressed genes. This was achieved by feeding IDs and expression values of those genes that had the words “transcription factor” appearing in their GO information as the seed for the network architecture, and the same data from the remaining differentially expressed genes as the data set from which to extract the network. Finally, the network was exported as comma-separated-value data sheets and imported to Cytoscape^61^ for visualization and comparative analysis of our inferred architecture against established biological pathways. Functional enrichment analysis of differentially expressed genes for GO biological process terms databases was performed using METASCAPE.^62^

### qPCR

To validate observed transcriptional differences in microarray analysis, qPCR analysis was performed. Tissue RNA samples were reverse transcribed into complementary DNA (cDNA) using the iScript cDNA synthesis kit (Bio-Rad, Hercules, CA). qPCR reactions were performed using IQ SYBR Green Supermix (Bio-Rad). cDNA was amplified using a 3-step program (40 cycles) with a MyiQ system (Bio-Rad). Gene expression levels were determined using iQ5 software using a delta cycle-treshold (ΔCt) relative quantification model. The geometric mean of *RPLP0* and *CYPA* was used as a normalization factor.

For gene expression analysis in intestinal organoids, the cells in Geltrex were lysed using TRI reagent (Sigma-Aldrich) and RNA was isolated according to the manufacturer’s instructions. Synthesis of cDNA was performed using the SensiFast cDNA Synthesis kit (Bioline GmbH, Luckenwalde, Germany). qPCR analysis was performed on the LightCycler480 (Roche, Mannheim, Germany) using the SensiMix SYBR Hi-Rox kit (Bioline GmbH) for amplification. Data were processed using LinRegPCR software (version 2016.1).^63^ The geometric mean of reference genes *B2MG* and *ACTB* was used for normalization. All primer sequences are provided in Supplementary Table 7.

### Apoptosis Assay

For apoptosis assays, organoids were plated in 10 μL Geltrex (20,000 cells/well) in a 96-well plate. Caspase-Glo 3/7 assay (Promega) was performed according to the manufacturer’s protocol. Luminescence was measured on the Spark Microplate Reader (Tecan, Männedorf, Switzerland). Data were corrected for background luminescence.

### Histology and Immunohistochemistry

Formalin-fixed tissue samples were embedded in paraffin and 4-μm sections were cut, deparaffinized in xylene, and rehydrated in graded ethanol to distilled water. Sections were stained with H&E for morphologic analysis. For immunohistochemical staining, endogenous peroxidase activity was blocked using 0.6% hydrogen peroxide in methanol for 15 minutes. Next, antigen retrieval was performed in 10 mmol/L citrate buffer (pH 6.0) at 90°C for 20 minutes. Nonspecific antibody binding was blocked with 5% bovine serum albumin. Sections then were incubated overnight at 4°C with the following primary antibodies: BiP (mouse, 1:1000; BD Biosciences, Franklin Lakes, NJ), XBP1s (mouse, 1:10,000; BioLegend, San Diego, CA), phospho-eIF2α (rabbit, 1:3000; Abcam, Cambridge, UK). The next day, sections were incubated with biotin-conjugated secondary antibodies (rabbit anti-mouse or swine anti-rabbit, 1:500; Dako, Glostrup, Denmark) for 60 minutes, followed by incubation with avidin-streptavidin complex (Vector Laboratories, Burlingame, CA) for 60 minutes. Antibody binding was visualized with 3,3’-diaminobenzidine (Dako), and sections were counterstained with hematoxylin.

### Transmission Electron Microscopy

Jejunal tissue exposed to ischemia-reperfusion from 2 patients was immersed in 3% glutaraldehyde fixative buffered in 0.09 mol/L KH_2_PO_4_ at pH 7.4. Next, samples were washed in 0.09 mol/L KH_2_PO_4_ buffer containing 7.5% sucrose and transferred to a 1% OsO_4_ fixative solution buffered with veronal acetate buffer (pH 7.4) plus 1.5% ferrocyanide. After washing in veronal acetate buffer plus 7% sucrose for 5 minutes at 4°C, dehydration was performed in graded ethanol series, followed by embedding in Epon (Burlington, VT). Tissue sections were examined with a Philips CM 100 electron microscope (Philips, Eindhoven, The Netherlands) at an accelerating voltage of 80 kV.

### Statistical Analysis

Statistical analysis of microarray data were performed in R as described in the paragraph Differential expression analysis. Statistical analysis of gene expression and apoptosis data was performed using GraphPad Prism version 6.01 (Graphpad software, San Diego, CA). These data were analyzed using the Kruskal–Wallis 1-way analysis of variance test followed by the Dunn post hoc test. *P* < .05 was considered significant.

All authors had access to the study data and reviewed and approved the final manuscript.

## CRediT Authorship Contributions

Anna Maria Kip (Conceptualization: Equal; Formal analysis: Equal; Funding acquisition: Equal; Investigation: Equal; Visualization: Equal; Writing – original draft: Lead; Organoid methodology and investigation: Lead)

Joep Grootjans (Investigation: Supporting; Writing – review & editing: Supporting)

Marco Manca (Formal analysis: Equal; Writing – review & editing: Supporting)

M'hamed Hadfoune (Investigation: Supporting)

Bas Boonen (Investigation: Supporting)

Joep P.M. Derikx (Investigation: Supporting; Writing – review & editing: Supporting)

Erik A.L. Biessen (Writing – review & editing: Supporting)

Steven W.M. Olde Damink (Supervision: Supporting; Writing – review & editing: Supporting)

Cornelis H.C. Dejong (Investigation: Supporting; Writing – review & editing: Supporting)

Wim A Buurman (Supervision: Supporting)

Kaatje Lenaerts (Conceptualization: Equal; Formal analysis: Equal; Funding acquisition: Equal; Supervision: Lead; Visualization: Equal; Writing – review & editing: Lead)
