# Supplementary material for: Temporal Transcript Profiling Identifies a Role for Unfolded Protein Stress in Human Gut Ischemia-Reperfusion Injury
Source: Cell Mol Gastroenterol Hepatol. 2021 Nov 11;13(3):681–94. doi: 10.1016/j.jcmgh.2021.11.001 (PMC8761776; doi:10.1016/j.jcmgh.2021.11.001)
Supplement: Supplementary Table [file mmc1.pdf]

Table S1. RNA samples included for gene expression analysis by Illumina Beadchips. Samples were randomized over three Beadchips.

Patient	Age	Sex	Condition	RIN	Chip	Array
1	72	male	30I/0R	8,6	4811636083	A
			30I/30R	8,2	4811636063	B
			30I/120R	8,8	4811636063	D
			C	7,3	4811636063	J
2	62	female	30I/0R	9,3	4811636083	D
			30I/30R	8,4	4811636083	H
			30I/120R	7,4	4811636083	I
			C	8,1	4811636083	K
3	73	male	30I/0R	9,2	4811636065	K
			30I/30R	8,8	4811636083	G
			30I/120R	7,3	4811636065	C
			C	8,1	4811636063	F
4	65	male	45I/0R	9,1	4811636065	I
			45I/30R	9,2	4811636063	A
			45I/120R	9,1	4811636083	C
			C	8,3	4811636065	A
5	54	female	45I/0R	9,4	4811636063	C
			45I/30R	9,6	4811636083	J
			45I/120R	8,5	4811636065	B
			C	7,1	4811636065	J
6	79	female	45I/0R	8,8	4811636065	L
			45I/30R	7,6	4811636083	L
			45I/120R	8,7	4811636063	L
			C	8,8	4811636065	H
7	76	female	45I/0R	8,9	4811636065	E
			45I/30R	8,4	4811636065	F
			45I/120R	7,8	4811636083	F
			C	8,7	4811636065	G

Table S2. Gene expression changes in the human intestine in response to ischemia-reperfusion. The 50 highest significantly up- and downregulated genes per condition (0R, 30R and 120R vs C) are listed.

Entrez ID	Gene Symbol	Gene Name	Fold change	Adjusted *P*-value
**0R vs C**			**Up**	
4069	*LYZ*	lysozyme (renal amyloidosis)	4,11	0,0327
83998	*REG4*	regenerating islet-derived family, member 4	3,37	0,0233
2353	*FOS*	v-fos FBJ murine osteosarcoma viral oncogene homolog	2,96	0,0494
27299	*ADAMDEC1*	ADAM-like, decysin 1	2,06	0,0488
642817	*LOC642817*	hypothetical LOC642817	1,88	0,0322
7494	*XBP1*	X-box binding protein 1	1,86	0,0258
7494	*XBP1*	X-box binding protein 1	1,85	0,0328
71	*ACTG1*	actin, gamma 1	1,84	0,0448
6175	*RPLP0*	ribosomal protein, large, P0 pseudogene 2	1,81	0,0336
10935	*PRDX3*	peroxiredoxin 3	1,80	0,0407
58505	*DC2*	oligosaccharyltransferase complex subunit; similar to DC2 protein	1,78	0,0181
64231	*MS4A6A*	membrane-spanning 4-domains, subfamily A, member 6A	1,69	0,0289
3150	*HMGN1*	high-mobility group nucleosome binding domain 1	1,64	0,0392
10175	*CNIH*	cornichon homolog (Drosophila)	1,63	0,0276
708	*C1QBP*	complement component 1, q subcomponent binding protein	1,63	0,0322
1109	*AKR1C4*	aldo-keto reductase family 1, member C4	1,63	0,0414
54504	*CPVL*	carboxypeptidase, vitellogenic-like	1,62	0,0407
114908	*TMEM123*	transmembrane protein 123	1,58	0,0335
10175	*CNIH*	cornichon homolog (Drosophila)	1,56	0,0182
6558	*SLC12A2*	solute carrier family 12 (sodium/potassium/chloride transporters), member 2	1,55	0,0276
6175	*RPLP0*	ribosomal protein, large, P0 pseudogene 2	1,55	0,0322
6613	*SUMO2*	SMT3 suppressor of mif two 3 homolog 2 (S. cerevisiae) pseudogene	1,54	0,0422
51522	*TMEM14C*	transmembrane protein 14C	1,53	0,0392
6218	*RPS17*	ribosomal protein S17	1,53	0,0182
2938	*GSTA1*	glutathione S-transferase alpha 1	1,53	0,0328
10252	*SPRY1*	sprouty homolog 1, antagonist of FGF signaling (Drosophila)	1,53	0,0442
2171	*FABP5*	fatty acid binding protein 5-like 2	1,53	0,0314
646567	*OSTCP2*	oligosaccharyltransferase complex subunit pseudogene 2	1,52	0,0449
11222	*MRPL3*	mitochondrial ribosomal protein L3	1,52	0,0372
515	*ATP5F1*	ATP synthase, H+ transporting, mitochondrial F0 complex, subunit B1	1,51	0,0470
6636	*SNRPF*	small nuclear ribonucleoprotein polypeptide F	1,51	0,0244
9403	*sep-15*	15 kDa selenoprotein	1,50	0,0130
7857	*SCG2*	secretogranin II (chromogranin C)	1,50	0,0375
51495	*PTPLAD1*	protein tyrosine phosphatase-like A domain containing 1	1,50	0,0068
10606	*PAICS*	phosphoribosylaminoimidazole carboxylase	1,50	0,0448
51192	*CKLF*	chemokine-like factor	1,50	0,0328
9204	*ZMYM6*	hypothetical LOC100130633; zinc finger, MYM-type 6	1,50	0,0103
52	*ACP1*	acid phosphatase 1, soluble	1,49	0,0392
84233	*TMEM126A*	transmembrane protein 126A	1,48	0,0182
6187	*RPS2*	ribosomal protein S2 pseudogene 8	1,47	0,0340
10412	*TINP1*	similar to TGF beta-inducible nuclear protein 1	1,47	0,0289
8804	*CREG1*	cellular repressor of E1A-stimulated genes 1	1,46	0,0197
8813	*DPM1*	dolichyl-phosphate mannosyltransferase polypeptide 1, catalytic subunit	1,46	0,0303
3094	*HINT1*	histidine triad nucleotide binding protein 1	1,45	0,0431
6727	*SRP14*	signal recognition particle 14kDa (homologous Alu RNA binding protein) pseud	1,45	0,0285
51014	*TMED7*	transmembrane emp24 protein transport domain containing 7	1,45	0,0197
6210	*RPS15A*	ribosomal protein S15a pseudogene 17	1,45	0,0289
54148	*MRPL39*	mitochondrial ribosomal protein L39	1,45	0,0182
86	*ACTL6A*	actin-like 6A	1,44	0,0233
9685	*CLINT1*	clathrin interactor 1	1,44	0,0499
**0R vs C**			**Down**	
126205	*NLRP8*	NLR family, pyrin domain containing 8	-1,88	0,0455
10299	*MARCH6*	membrane-associated ring finger (C3HC4) 6	-1,72	0,0328
729603	*LOC729603*	calcium binding protein P22 pseudogene	-1,68	0,0494
1577	*CYP3A5*	cytochrome P450, family 3, subfamily A, polypeptide 5	-1,63	0,0372
136051	*ZNF786*	zinc finger protein 786	-1,61	0,0427
8566	*PDXK*	pyridoxal (pyridoxine, vitamin B6) kinase	-1,59	0,0431
9679	*FAM53B*	family with sequence similarity 53, member B	-1,57	0,0276
79784	*MYH14*	myosin, heavy chain 14	-1,55	0,0292
7430	*EZR*	ezrin	-1,53	0,0499
4967	*OGDH*	oxoglutarate (alpha-ketoglutarate) dehydrogenase (lipoamide)	-1,53	0,0335
3691	*ITGB4*	integrin, beta 4	-1,51	0,0322

55667	*DENND4C*	DENN/MADD domain containing 4C	-1,51	0,0182
192683	*SCAMP5*	secretory carrier membrane protein 5	-1,48	0,0442
10908	*PNPLA6*	patatin-like phospholipase domain containing 6	-1,46	0,0322
5119	*CHMP1A*	chromatin modifying protein 1A	-1,45	0,0182
11282	*MGAT4B*	mannosyl (alpha-1,3-)-glycoprotein beta-1,4-N-acetylglucosaminyltransferase,	-1,45	0,0328
8073	*PTP4A2*	protein tyrosine phosphatase type IVA, member 2	-1,45	0,0053
4296	*MAP3K11*	mitogen-activated protein kinase kinase kinase 11	-1,45	0,0133
23140	*ZZEF1*	zinc finger, ZZ-type with EF-hand domain 1	-1,44	0,0096
79157	*MFSD11*	major facilitator superfamily domain containing 11	-1,44	0,0289
5753	*PTK6*	PTK6 protein tyrosine kinase 6	-1,43	0,0455
90007	*MIDN*	midnolin	-1,43	0,0328
23277	*KIAA0664*	KIAA0664	-1,42	0,0276
9600	*PITPNM1*	phosphatidylinositol transfer protein, membrane-associated 1	-1,42	0,0392
728294	*D2HGDH*	D-2-hydroxyglutarate dehydrogenase	-1,41	0,0444
25920	*COBRA1*	cofactor of BRCA1	-1,41	0,0258
9927	*MFN2*	mitofusin 2	-1,41	0,0278
6687	*SPG7*	spastic paraplegia 7 (pure and complicated autosomal recessive)	-1,40	0,0328
10163	*WASF2*	WAS protein family, member 2	-1,40	0,0289
64780	*MICAL1*	microtubule associated monoxygenase, calponin and LIM domain containing 1	-1,40	0,0440
4248	*MGAT3*	mannosyl (beta-1,4-)-glycoprotein beta-1,4-N-acetylglucosaminyltransferase	-1,40	0,0372
3609	*ILF3*	interleukin enhancer binding factor 3, 90kDa	-1,39	0,0289
90639	*COX19*	COX19 cytochrome c oxidase assembly homolog (S. cerevisiae)	-1,39	0,0293
440704	*LOC440704*	hypothetical gene supported by BC042042	-1,39	0,0444
57464	*FAM40B*	family with sequence similarity 40, member B	-1,39	0,0412
3636	*INPPL1*	inositol polyphosphate phosphatase-like 1	-1,38	0,0289
64761	*PARP12*	poly (ADP-ribose) polymerase family, member 12	-1,38	0,0448
317762	*C14orf65*	coiled-coil domain containing 85C	-1,38	0,0236
79720	*VPS37B*	vacuolar protein sorting 37 homolog B (S. cerevisiae)	-1,38	0,0303
8408	*ULK1*	unc-51-like kinase 1 (C. elegans)	-1,38	0,0289
9807	*IHPK1*	inositol hexakisphosphate kinase 1	-1,37	0,0258
23352	*UBR4*	ubiquitin protein ligase E3 component n-recognin 4	-1,37	0,0372
83734	*ATG10*	ATG10 autophagy related 10 homolog (S. cerevisiae)	-1,36	0,0182
5917	*RARS*	arginyl-tRNA synthetase	-1,36	0,0448
27072	*VPS41*	vacuolar protein sorting 41 homolog (S. cerevisiae)	-1,36	0,0375
140628	*GATA5*	GATA binding protein 5	-1,34	0,0462
2017	*CTTN*	cortactin	-1,34	0,0181
6829	*SUPT5H*	suppressor of Ty 5 homolog (S. cerevisiae)	-1,34	0,0292
22906	*TRAK1*	trafficking protein, kinesin binding 1	-1,34	0,0328
79784	*MYH14*	myosin, heavy chain 14	-1,33	0,0289

**30R vs C**			**Up**	
2353	*FOS*	v-fos FBJ murine osteosarcoma viral oncogene homolog	20,89	0,0000
2354	*FOSB*	FBJ murine osteosarcoma viral oncogene homolog B	16,64	0,0000
3304	*HSPA1B*	heat shock 70kDa protein 1A; heat shock 70kDa protein 1B	14,33	0,0000
3303	*HSPA1A*	heat shock 70kDa protein 1A; heat shock 70kDa protein 1B	13,53	0,0000
1843	*DUSP1*	dual specificity phosphatase 1	10,40	0,0000
3725	*JUN*	jun oncogene	9,53	0,0000
467	*ATF3*	activating transcription factor 3	9,44	0,0000
1958	*EGR1*	early growth response 1	9,04	0,0000
7832	*BTG2*	BTG family, member 2	7,51	0,0000
3337	*DNAJB1*	DnaJ (Hsp40) homolog, subfamily B, member 1	6,97	0,0001
1839	*HBEGF*	heparin-binding EGF-like growth factor	6,66	0,0000
5996	*RGS1*	regulator of G-protein signaling 1	5,45	0,0000
4929	*NR4A2*	nuclear receptor subfamily 4, group A, member 2	5,07	0,0000
7538	*ZFP36*	zinc finger protein 36, C3H type, homolog (mouse)	4,92	0,0000
23645	*PPP1R15A*	protein phosphatase 1, regulatory (inhibitor) subunit 15A	4,88	0,0000
10365	*KLF2*	Kruppel-like factor 2 (lung)	4,86	0,0001
3491	*CYR61*	cysteine-rich, angiogenic inducer, 61	3,92	0,0000
1490	*CTGF*	connective tissue growth factor	3,35	0,0013
3320	*HSP90AA1*	heat shock protein 90kDa alpha (cytosolic), class A member 2	3,22	0,0000
414062	*CCL3L3*	chemokine (C-C motif) ligand 3-like 3	3,19	0,0001
64651	*AXUD1*	cysteine-serine-rich nuclear protein 1	3,17	0,0000
1490	*CTGF*	connective tissue growth factor	3,15	0,0023
6515	*SLC2A3*	solute carrier family 2 (facilitated glucose transporter), member 3	3,01	0,0001
1316	*KLF6*	Kruppel-like factor 6	2,95	0,0001
8870	*IER3*	immediate early response 3	2,91	0,0000
388	*RHOB*	ras homolog gene family, member B	2,90	0,0001
10808	*HSPH1*	heat shock 105kDa/110kDa protein 1	2,84	0,0064
90637	*ZFAND2A*	zinc finger, AN1-type domain 2A	2,84	0,0209
969	*CD69*	CD69 molecule	2,79	0,0001

5743	*PTGS2*	prostaglandin-endoperoxide synthase 2 (prostaglandin G/H synthase and cycl	2,70	0,0165
10221	*TRIB1*	tribbles homolog 1 (Drosophila)	2,68	0,0000
9314	*KLF4*	Kruppel-like factor 4 (gut)	2,68	0,0002
9531	*BAG3*	BCL2-associated athanogene 3	2,66	0,0053
9023	*CH25H*	cholesterol 25-hydroxylase	2,63	0,0002
56892	*C8orf4*	chromosome 8 open reading frame 4	2,60	0,0027
5552	*SRGN*	serglycin	2,55	0,0113
8553	*BHLHB2*	basic helix-loop-helix family, member e40	2,55	0,0000
6355	*CCL8*	chemokine (C-C motif) ligand 8	2,53	0,0019
1164	*CKS2*	CDC28 protein kinase regulatory subunit 2	2,53	0,0268
3399	*ID3*	inhibitor of DNA binding 3, dominant negative helix-loop-helix protein	2,51	0,0007
3726	*JUNB*	jun B proto-oncogene	2,50	0,0000
3336	*HSPE1*	heat shock 10kDa protein 1 (chaperonin 10)	2,42	0,0014
1847	*DUSP5*	dual specificity phosphatase 5	2,38	0,0019
8848	*TSC22D1*	TSC22 domain family, member 1	2,36	0,0015
3315	*HSPB1*	heat shock 27kDa protein-like 2 pseudogene; heat shock 27kDa protein 1	2,31	0,0113
3490	*IGFBP7*	insulin-like growth factor binding protein 7	2,26	0,0299
10124	*ARL4A*	ADP-ribosylation factor-like 4A	2,23	0,0053
3301	*DNAJA1*	DnaJ (Hsp40) homolog, subfamily A, member 1	2,17	0,0027
4953	*ODC1*	ornithine decarboxylase 1	2,16	0,0001
114789	*SLC25A25*	solute carrier family 25 (mitochondrial carrier; phosphate carrier), member 25	2,12	0,0005

**30R vs C**			**Down**	
89872	*AQP10*	aquaporin 10	-3,37	0,0424
4680	*CEACAM6*	carcinoembryonic antigen-related cell adhesion molecule 6 (non-specific cross	-3,32	0,0148
1510	*CTSE*	cathepsin E	-2,56	0,0245
5265	*SERPINA1*	serpin peptidase inhibitor, clade A (alpha-1 antiproteinase, antitrypsin), membe	-2,41	0,0304
8566	*PDXK*	pyridoxal (pyridoxine, vitamin B6) kinase	-2,28	0,0001
54825	*PCLKC*	protocadherin 24	-2,23	0,0063
1687	*DFNA5*	deafness, autosomal dominant 5	-2,16	0,0165
29881	*NPC1L1*	NPC1 (Niemann-Pick disease, type C1, gene)-like 1	-2,00	0,0223
6564	*SLC15A1*	solute carrier family 15 (oligopeptide transporter), member 1	-1,93	0,0099
1308	*COL17A1*	collagen, type XVII, alpha 1	-1,90	0,0030
59272	*ACE2*	angiotensin I converting enzyme (peptidyl-dipeptidase A) 2	-1,87	0,0165
4967	*OGDH*	oxoglutarate (alpha-ketoglutarate) dehydrogenase (lipoamide)	-1,83	0,0012
629	*CFB*	complement factor B	-1,83	0,0154
733	*C8G*	complement component 8, gamma polypeptide	-1,81	0,0469
3914	*LAMB3*	laminin, beta 3	-1,81	0,0304
6337	*SCNN1A*	sodium channel, nonvoltage-gated 1 alpha	-1,80	0,0100
5243	*ABCB1*	ATP-binding cassette, sub-family B (MDR/TAP), member 1	-1,79	0,0272
246181	*AFAR3*	aldo-keto reductase family 7-like	-1,78	0,0227
2875	*GPT*	glutamic-pyruvate transaminase (alanine aminotransferase)	-1,78	0,0053
338	*APOB*	apolipoprotein B (including Ag(x) antigen)	-1,78	0,0289
55715	*DOK4*	docking protein 4	-1,77	0,0038
347741	*OTOP3*	otopetrin 3	-1,76	0,0196
192683	*SCAMP5*	secretory carrier membrane protein 5	-1,76	0,0019
79762	*C1orf115*	chromosome 1 open reading frame 115	-1,74	0,0255
25845	*LOC25845*	hypothetical LOC25845	-1,74	0,0427
11181	*TREH*	trehalase (brush-border membrane glycoprotein)	-1,71	0,0185
164091	*PAQR7*	progestin and adipoQ receptor family member VII	-1,70	0,0219
3938	*LCT*	lactase	-1,70	0,0368
11282	*MGAT4B*	mannosyl (alpha-1,3-)-glycoprotein beta-1,4-N-acetylglucosaminyltransferase,	-1,69	0,0012
535	*ATP6V0A1*	ATPase, H+ transporting, lysosomal V0 subunit a1	-1,69	0,0055
53841	*MUPCDH*	mucin-like protocadherin	-1,68	0,0434
7430	*EZR*	hypothetical protein LOC100129652; ezrin	-1,68	0,0122
4248	*MGAT3*	mannosyl (beta-1,4-)-glycoprotein beta-1,4-N-acetylglucosaminyltransferase	-1,67	0,0005
399665	*FAM102A*	family with sequence similarity 102, member A	-1,67	0,0278
54566	*EPB41L4B*	erythrocyte membrane protein band 4.1 like 4B	-1,66	0,0190
6462	*SHBG*	sex hormone-binding globulin	-1,66	0,0370
6813	*STXBP2*	syntaxin binding protein 2	-1,66	0,0025
9679	*FAM53B*	family with sequence similarity 53, member B	-1,65	0,0038
3691	*ITGB4*	integrin, beta 4	-1,65	0,0038
83715	*ESPN*	espin	-1,65	0,0240
10223	*GPA33*	glycoprotein A33 (transmembrane)	-1,64	0,0304
7512	*XPNPEP2*	X-prolyl aminopeptidase (aminopeptidase P) 2, membrane-bound	-1,62	0,0490
10908	*PNPLA6*	patatin-like phospholipase domain containing 6	-1,61	0,0031
79065	*ATG9A*	ATG9 autophagy related 9 homolog A (S. cerevisiae)	-1,60	0,0095
4354	*MPP1*	membrane protein, palmitoylated 1, 55kDa	-1,60	0,0271
55884	*WSB2*	WD repeat and SOCS box-containing 2	-1,60	0,0041
6523	*SLC5A1*	solute carrier family 5 (sodium/glucose cotransporter), member 1	-1,58	0,0171

6653	*SORL1*	sortilin-related receptor, L(DLR class) A repeats-containing	-1,57	0,0239
11148	*HHLA2*	HERV-H LTR-associating 2	-1,56	0,0370
4311	*MME*	membrane metallo-endopeptidase	-1,56	0,0304

**120R vs C**			**Up**	
3303	*HSPA1A*	heat shock 70kDa protein 1A; heat shock 70kDa protein 1B	12,19	0,0000
2354	*FOSB*	FBJ murine osteosarcoma viral oncogene homolog B	7,93	0,0001
10808	*HSPH1*	heat shock 105kDa/110kDa protein 1	7,12	0,0000
3304	*HSPA1B*	heat shock 70kDa protein 1A; heat shock 70kDa protein 1B	6,73	0,0002
1604	*CD55*	CD55 molecule, decay accelerating factor for complement (Cromer blood grou	5,73	0,0000
3320	*HSP90AA1*	heat shock protein 90kDa alpha (cytosolic), class A member 2; heat shock pro	4,45	0,0000
9531	*BAG3*	BCL2-associated athanogene 3	4,12	0,0001
3337	*DNAJB1*	DnaJ (Hsp40) homolog, subfamily B, member 1	4,06	0,0013
1958	*EGR1*	early growth response 1	4,06	0,0003
3949	*LDLR*	low density lipoprotein receptor	3,98	0,0001
6280	*S100A9*	S100 calcium binding protein A9	3,93	0,0250
6279	*S100A8*	S100 calcium binding protein A8	3,86	0,0225
2810	*SFN*	stratifin	3,64	0,0001
90637	*ZFAND2A*	zinc finger, AN1-type domain 2A	3,46	0,0027
5552	*SRGN*	serglycin	3,30	0,0006
1847	*DUSP5*	dual specificity phosphatase 5	3,26	0,0000
3336	*HSPE1*	heat shock 10kDa protein 1 (chaperonin 10)	3,19	0,0000
3301	*DNAJA1*	DnaJ (Hsp40) homolog, subfamily A, member 1	3,16	0,0000
1839	*HBEGF*	heparin-binding EGF-like growth factor	3,11	0,0014
5209	*PFKFB3*	6-phosphofructo-2-kinase/fructose-2,6-biphosphatase 3	3,06	0,0016
6574	*SLC20A1*	solute carrier family 20 (phosphate transporter), member 1	3,02	0,0000
8870	*IER3*	immediate early response 3	3,02	0,0000
23645	*PPP1R15A*	protein phosphatase 1, regulatory (inhibitor) subunit 15A	3,01	0,0000
64651	*AXUD1*	cysteine-serine-rich nuclear protein 1	3,01	0,0000
467	*ATF3*	activating transcription factor 3	3,00	0,0122
3329	*HSPD1*	heat shock 60kDa protein 1 (chaperonin) pseudogene 5	2,99	0,0001
3638	*INSIG1*	insulin induced gene 1	2,92	0,0033
6515	*SLC2A3*	solute carrier family 2 (facilitated glucose transporter), member 3	2,91	0,0001
6347	*CCL2*	chemokine (C-C motif) ligand 2	2,88	0,0225
4953	*ODC1*	ornithine decarboxylase 1	2,78	0,0000
4929	*NR4A2*	nuclear receptor subfamily 4, group A, member 2	2,76	0,0021
5743	*PTGS2*	prostaglandin-endoperoxide synthase 2 (prostaglandin G/H synthase and cycl	2,76	0,0065
83716	*CRISPLD2*	cysteine-rich secretory protein LCCL domain containing 2	2,71	0,0014
1051	*CEBPB*	CCAAT/enhancer binding protein (C/EBP), beta	2,71	0,0000
10221	*TRIB1*	tribbles homolog 1 (Drosophila)	2,71	0,0000
55466	*DNAJA4*	DnaJ (Hsp40) homolog, subfamily A, member 4	2,71	0,0006
7538	*ZFP36*	zinc finger protein 36, C3H type, homolog (mouse)	2,69	0,0021
2012	*EMP1*	epithelial membrane protein 1	2,69	0,0001
3320	*HSP90AA1*	heat shock protein 90kDa alpha (cytosolic), class A member 2; heat shock pro	2,68	0,0000
3315	*HSPB1*	heat shock 27kDa protein-like 2 pseudogene	2,66	0,0014
10365	*KLF2*	Kruppel-like factor 2 (lung)	2,65	0,0058
5552	*SRGN*	serglycin	2,59	0,0023
1843	*DUSP1*	dual specificity phosphatase 1	2,57	0,0047
301	*ANXA1*	annexin A1	2,53	0,0012
3336	*HSPE1*	heat shock 10kDa protein 1 (chaperonin 10)	2,50	0,0001
27314	*RAB30*	RAB30, member RAS oncogene family	2,50	0,0001
29950	*SERTAD1*	SERTA domain containing 1	2,49	0,0000
4071	*TM4SF1*	transmembrane 4 L six family member 1	2,47	0,0001
3725	*JUN*	jun oncogene	2,45	0,0118
5328	*PLAU*	plasminogen activator, urokinase	2,43	0,0001

**120R vs C**			**Down**	
89872	*AQP10*	aquaporin 10	-4,38	0,0071
1510	*CTSE*	cathepsin E	-3,12	0,0028
2981	*GUCA2B*	guanylate cyclase activator 2B (uroguanylin)	-2,81	0,0267
4680	*CEACAM6*	carcinoembryonic antigen-related cell adhesion molecule 6 (non-specific cross	-2,77	0,0163
5265	*SERPINA1*	serpin peptidase inhibitor, clade A (alpha-1 antiproteinase, antitrypsin), membe	-2,67	0,0076
345	*APOC3*	apolipoprotein C-III	-2,62	0,0135
5265	*SERPINA1*	serpin peptidase inhibitor, clade A (alpha-1 antiproteinase, antitrypsin), membe	-2,57	0,0117
1687	*DFNA5*	deafness, autosomal dominant 5	-2,55	0,0016
79170	*ATAD4*	ATPase family, AAA domain containing 4	-2,53	0,0009
339221	*ENPP7*	ectonucleotide pyrophosphatase/phosphodiesterase 7	-2,41	0,0222
79762	*C1orf115*	chromosome 1 open reading frame 115	-2,37	0,0003
51471	*NAT8B*	N-acetyltransferase 8 (GCN5-related, putative); N-acetyltransferase 8B (GCN	-2,36	0,0108
339221	*ENPP7*	ectonucleotide pyrophosphatase/phosphodiesterase 7	-2,31	0,0307

1576	*CYP3A4*	cytochrome P450, family 3, subfamily A, polypeptide 4	-2,29	0,0082
1510	*CTSE*	cathepsin E	-2,21	0,0131
5169	*ENPP3*	ectonucleotide pyrophosphatase/phosphodiesterase 3	-2,14	0,0096
5210	*PFKFB4*	6-phosphofructo-2-kinase/fructose-2,6-biphosphatase 4	-2,12	0,0019
1559	*CYP2C9*	cytochrome P450, family 2, subfamily C, polypeptide 9	-2,08	0,0218
53354	*PANK1*	pantothenate kinase 1	-2,07	0,0020
1124	*CHN2*	chimerin (chimaerin) 2	-2,02	0,0015
8566	*PDXK*	pyridoxal (pyridoxine, vitamin B6) kinase	-2,02	0,0006
733	*C8G*	complement component 8, gamma polypeptide	-2,00	0,0109
4094	*MAF*	v-maf musculoaponeurotic fibrosarcoma oncogene homolog (avian)	-2,00	0,0098
140803	*TRPM6*	transient receptor potential cation channel, subfamily M, member 6	-1,99	0,0002
10	*NAT2*	N-acetyltransferase 2 (arylamine N-acetyltransferase)	-1,98	0,0019
54825	*PCLKC*	protocadherin 24	-1,97	0,0095
1030	*CDKN2B*	cyclin-dependent kinase inhibitor 2B (p15, inhibits CDK4)	-1,97	0,0005
1308	*COL17A1*	collagen, type XVII, alpha 1	-1,96	0,0009
5651	*PRSS7*	protease, serine, 7 (enterokinase)	-1,95	0,0369
25845	*LOC25845*	hypothetical LOC25845	-1,94	0,0078
948	*CD36*	CD36 molecule (thrombospondin receptor)	-1,93	0,0238
6564	*SLC15A1*	solute carrier family 15 (oligopeptide transporter), member 1	-1,92	0,0045
2065	*ERBB3*	v-erb-b2 erythroblastic leukemia viral oncogene homolog 3 (avian)	-1,92	0,0002
4225	*MEP1B*	meprin A, beta	-1,92	0,0148
2064	*ERBB2*	v-erb-b2 erythroblastic leukemia viral oncogene homolog 2, neuro/glioblastoma	-1,90	0,0003
56241	*SUSD2*	sushi domain containing 2	-1,89	0,0432
10720	*UGT2B11*	UDP glucuronosyltransferase 2 family, polypeptide B11	-1,89	0,0491
8743	*TNFSF10*	tumor necrosis factor (ligand) superfamily, member 10	-1,88	0,0004
164091	*PAQR7*	progestin and adipoQ receptor family member VII	-1,86	0,0033
246181	*AFAR3*	aldo-keto reductase family 7-like	-1,85	0,0067
6505	*SLC1A1*	solute carrier family 1 (neuronal/epithelial high affinity glutamate transporter, sy	-1,85	0,0014
7512	*XPNPEP2*	X-prolyl aminopeptidase (aminopeptidase P) 2, membrane-bound	-1,83	0,0074
192683	*SCAMP5*	secretory carrier membrane protein 5	-1,82	0,0005
59272	*ACE2*	angiotensin I converting enzyme (peptidyl-dipeptidase A) 2	-1,82	0,0096
11181	*TREH*	trehalase (brush-border membrane glycoprotein)	-1,80	0,0042
55715	*DOK4*	docking protein 4	-1,80	0,0014
4311	*MME*	membrane metallo-endopeptidase	-1,80	0,0021
3938	*LCT*	lactase	-1,80	0,0107
338	*APOB*	apolipoprotein B (including Ag(x) antigen)	-1,80	0,0127
29881	*NPC1L1*	NPC1 (Niemann-Pick disease, type C1, gene)-like 1	-1,80	0,0251

Table S3. Enrichment analysis of Gene Ontology terms for genes differentially expressed during human intestinal ischemia-reperfusion.

GOBPID	*P*-value	OddsRatio	ExpCount	Count	Size	Term
**0R to 30R**						
GO:0006986	4,9E-09	25,36	0,39	8	65	response to unfolded protein
GO:0051789	1,9E-13	24,36	0,70	13	116	response to protein stimulus
GO:0006916	1,6E-07	9,13	1,42	11	235	anti-apoptosis
GO:0009607	9,1E-08	6,54	2,78	15	459	response to biotic stimulus
GO:0010033	9,0E-13	7,13	5,39	27	889	response to organic substance
GO:0009628	4,4E-06	5,82	2,40	12	396	response to abiotic stimulus
GO:0051726	2,8E-06	5,60	2,73	13	450	regulation of cell cycle
GO:0009605	1,7E-06	4,82	3,99	16	658	response to external stimulus
GO:0006915	5,2E-09	4,94	6,78	25	1118	apoptosis
GO:0012501	6,0E-09	4,90	6,82	25	1126	programmed cell death
GO:0042127	2,3E-06	4,28	5,12	18	845	regulation of cell proliferation
GO:0008283	4,7E-08	4,49	6,99	24	1154	cell proliferation
GO:0042981	4,4E-06	4,08	5,36	18	884	regulation of apoptosis
GO:0008219	3,7E-08	4,43	7,47	25	1233	cell death
GO:0016265	3,9E-08	4,41	7,49	25	1236	death
GO:0043067	5,0E-06	4,04	5,41	18	892	regulation of programmed cell death
GO:0042221	1,0E-09	4,71	9,03	30	1490	response to chemical stimulus
GO:0010941	5,6E-06	4,00	5,45	18	899	regulation of cell death
GO:0006950	4,4E-12	5,24	11,21	37	1849	response to stress
GO:0048523	5,8E-12	5,18	11,31	37	1866	negative regulation of cellular process
GO:0048519	1,6E-11	4,92	12,32	38	2033	negative regulation of biological process
GO:0048522	3,2E-07	3,48	12,24	31	2020	positive regulation of cellular process
GO:0048518	2,2E-07	3,45	13,45	33	2219	positive regulation of biological process
GO:0048513	3,7E-06	3,18	11,55	28	1906	organ development
GO:0050896	7,2E-11	4,29	21,84	50	3604	response to stimulus
GO:0048856	8,2E-06	2,82	16,49	34	2720	anatomical structure development
GO:0031323	1,8E-06	2,90	21,80	42	3597	regulation of cellular metabolic process
GO:0050794	1,5E-07	3,35	36,48	60	6018	regulation of cellular process
GO:0050789	4,7E-07	3,22	38,53	61	6357	regulation of biological process
GO:0065007	5,7E-06	2,87	40,91	61	6749	biological regulation
**30R to 120R**						
GO:0048514	5,5E-06	3,64	5,76	19	276	blood vessel morphogenesis
GO:0001525	1,1E-04	3,36	4,85	15	232	angiogenesis
GO:0032535	3,8E-06	3,35	7,25	22	347	regulation of cellular component size
GO:0001944	7,3E-06	3,32	6,98	21	334	vasculature development
GO:0001568	1,6E-05	3,24	6,77	20	324	blood vessel development
GO:0007264	2,9E-05	2,84	8,86	23	424	small GTPase mediated signal transduction
GO:0090066	5,3E-05	2,79	8,58	22	411	regulation of anatomical structure size
GO:0051128	2,1E-05	2,47	13,81	31	661	regulation of cellular component organization
GO:0006366	3,3E-05	2,20	19,01	38	910	transcription from RNA polymerase II promoter
GO:0006915	6,4E-06	2,21	23,35	46	1118	apoptosis
GO:0012501	7,7E-06	2,19	23,52	46	1126	programmed cell death
GO:0008219	7,9E-06	2,13	25,75	49	1233	cell death
GO:0016265	8,4E-06	2,13	25,81	49	1236	death
GO:0048518	5,7E-09	2,23	46,34	85	2219	positive regulation of biological process
GO:0042221	3,5E-05	1,94	31,12	54	1490	response to chemical stimulus
GO:0048522	1,0E-06	2,02	42,19	73	2020	positive regulation of cellular process
GO:0007165	3,8E-05	1,79	45,80	72	2193	signal transduction
GO:0023060	2,5E-05	1,77	52,76	81	2526	signal transmission
GO:0023033	3,0E-05	1,76	52,19	80	2499	signaling pathway
GO:0023046	2,7E-05	1,77	52,88	81	2532	signaling process
GO:0051171	5,0E-05	1,70	60,71	89	2907	regulation of nitrogen compound metabolic process
GO:0019219	6,2E-05	1,69	60,19	88	2882	regulation of nucleobase, nucleoside, nucleotide and nucleic acid metabolic process
GO:0050794	4,5E-12	2,31	125,69	183	6018	regulation of cellular process
GO:0031323	1,9E-05	1,70	75,12	107	3597	regulation of cellular metabolic process
GO:0050789	1,0E-11	2,29	132,77	189	6357	regulation of biological process
GO:0023052	2,9E-05	1,68	73,14	104	3502	signaling
GO:0080090	5,1E-05	1,66	71,36	101	3417	regulation of primary metabolic process
GO:0060255	9,6E-05	1,64	67,98	96	3255	regulation of macromolecule metabolic process
GO:0065007	5,1E-11	2,24	140,95	195	6749	biological regulation
GO:0019222	4,6E-05	1,64	81,16	112	3886	regulation of metabolic process

**0R to 30R**						
GO:0003690	2,1E-04	10,24	0,54	5	96	double-stranded DNA binding
GO:0031072	8,9E-04	10,23	0,43	4	76	heat shock protein binding
GO:0051082	3,6E-04	9,04	0,61	5	108	unfolded protein binding
GO:0016564	1,8E-05	6,03	1,89	10	335	transcription repressor activity
GO:0003700	5,0E-09	5,48	5,22	22	925	sequence-specific DNA binding transcription factor activity
GO:0016563	2,7E-05	5,21	2,42	11	428	transcription activator activity
GO:0043565	1,5E-06	5,17	3,46	15	613	sequence-specific DNA binding
GO:0008134	3,1E-05	4,74	2,91	12	516	transcription factor binding
GO:0030528	7,4E-07	4,47	5,25	19	930	transcription regulator activity
GO:0046983	9,6E-05	4,18	3,28	12	581	protein dimerization activity
GO:0005515	3,0E-05	2,70	44,83	63	7945	protein binding
**30R to 120R**						
GO:0003688	2,6E-04	37,01	0,14	3	7	DNA replication origin binding
GO:0030291	2,2E-05	19,10	0,36	5	18	protein serine/threonine kinase inhibitor activity
GO:0004860	8,1E-04	7,75	0,74	5	37	protein kinase inhibitor activity
GO:0050839	8,1E-04	7,75	0,74	5	37	cell adhesion molecule binding
GO:0003924	3,7E-08	5,21	4,15	19	207	GTPase activity
GO:0003714	1,5E-04	4,19	2,87	11	143	transcription corepressor activity
GO:0005525	6,0E-08	3,93	7,16	25	357	GTP binding
GO:0019001	1,1E-07	3,79	7,40	25	369	guanyl nucleotide binding
GO:0032561	1,1E-07	3,79	7,40	25	369	guanyl ribonucleotide binding
GO:0016564	1,5E-04	2,89	6,72	18	335	transcription repressor activity
GO:0003712	9,3E-04	2,52	7,18	17	358	transcription cofactor activity
GO:0030528	5,1E-05	2,17	18,65	37	930	transcription regulator activity
GO:0005515	2,6E-06	1,76	159,34	198	7945	protein binding

GOBPID — *Gene Ontology Biological Process Identification number*
GOMFID — *Gene Ontology Molecular Function Identification number*
Pvalue — *p value given by the hypergeometric test (P<0.01)*
OddsRatio — *ratio of odds that a GO term is enriched in the selected category*
ExpCount — *expected number of transcripts found associated with the GO term for enrichment*
Count — *real number of transcripts found associated with the GO term*
Size — *population size of transcripts found associated with the GO term within the analysis*
Term — *Gene Ontology description term*

Table S4. KEGG pathway analysis for genes differentially expressed during human ischemia-reperfusion

KEGG ID	Pathway name	Adjusted P-value	Size	Count	Status
**30R vs 0R**					
4010	MAPK signaling pathway	1,92E-06	263	12	Inhibited
4141	Protein processing in endoplasmic reticulum	5,43E-06	161	8	Inhibited
5140	Leishmaniasis	3,34E-02	71	4	Inhibited
5120	Epithelial cell signaling in Helicobacter pylori infection	3,34E-02	67	3	Activated
4621	NOD-like receptor signaling pathway	4,67E-02	62	3	Inhibited
4912	GnRH signaling pathway	4,67E-02	98	2	Activated
5210	Colorectal cancer	4,67E-02	62	2	Activated
5142	Chagas disease	4,67E-02	104	4	Inhibited
**120R vs 30R**					
5130	Pathogenic Escherichia coli infection	1,01E-02	57	7	Activated
4110	Cell cycle	1,01E-02	124	9	Inhibited
5100	Bacterial invasion of epithelial cells	1,01E-02	71	7	Activated
5210	Colorectal cancer	1,01E-02	62	4	Inhibited
4010	MAPK signaling pathway	1,01E-02	263	14	Activated
4350	TGF-beta signaling pathway	1,29E-02	84	8	Activated
4920	Adipocytokine signaling pathway	1,76E-02	67	5	Activated
5222	Small cell lung cancer	1,76E-02	84	7	Activated
5416	Viral myocarditis	2,89E-02	70	3	Activated
5014	Amyotrophic lateral sclerosis	2,89E-02	53	4	Activated
4810	Regulation of actin cytoskeleton	2,89E-02	210	10	Activated
5131	Shigellosis	4,24E-02	62	5	Activated
**Overall**					
5130	Pathogenic Escherichia coli infection	6,43E-06	57	21	Activated
4141	Protein processing in endoplasmic reticulum	2,95E-03	161	32	Inhibited
4012	ErbB signaling pathway	3,59E-03	87	19	Inhibited
5223	Non-small cell lung cancer	4,88E-03	54	13	Inhibited
3320	PPAR signaling pathway	4,88E-03	69	17	Inhibited
5211	Renal cell carcinoma	9,57E-03	70	13	Inhibited
4722	Neurotrophin signaling pathway	1,37E-02	126	24	Activated
5219	Bladder cancer	1,37E-02	42	11	Inhibited
4350	TGF-beta signaling pathway	1,54E-02	84	18	Inhibited
5020	Prion disease	1,54E-02	35	9	Inhibited
4010	MAPK signaling pathway	1,54E-02	263	40	Inhibited
5110	Vibrio cholerae infection	1,54E-02	53	12	Activated
5100	Bacterial invasion of epithelial cells	1,54E-02	71	15	Inhibited
4510	Focal adhesion	1,59E-02	198	28	Inhibited
5131	Shigellosis	1,59E-02	62	14	Inhibited
4370	VEGF signaling pathway	1,68E-02	74	13	Inhibited
4666	Fc gamma R-mediated phagocytosis	2,67E-02	93	17	Inhibited
4912	GnRH signaling pathway	2,80E-02	98	15	Inhibited
5210	Colorectal cancer	2,94E-02	62	10	Activated
4910	Insulin signaling pathway	2,94E-02	137	22	Inhibited
4142	Lysosome	4,37E-02	118	21	Inhibited

**No significant enrichment of KEGG pathways in the 0RvsC time-frame*

Count — *Number of differentially expressed genes in the KEGG pathway*

Size — *Total number of genes in the KEGG pathway*

Table S5. Transcription factors and corresponding number of up- and downregulated targets in the regulatory network

Entrez ID	Gene Symbol	Gene Name	Number of targets Up	Down
64651	*CSRNP1*	cysteine-serine-rich nuclear protein 1	1	0
159296	*NKX2-3*	NK2 homeobox 3	594	0
2000	*ELF4*	E74-like factor 4 (ets domain transcription factor)	489	0
3091	*HIF1A*	hypoxia inducible factor 1, alpha subunit (basic helix-loop-helix transcription factor)	353	148
4335	*MNT*	MAX binding protein	99	20
1052	*CEBPD*	CCAAT/enhancer binding protein (C/EBP), delta	76	0
2354	*FOSB*	FBJ murine osteosarcoma viral oncogene homolog B	67	0
3202	*HOXA5*	homeobox A5	64	6
1649	*DDIT3*	DNA-damage-inducible transcript 3	38	0
1958	*EGR1*	early growth response 1	26	0
9314	*KLF4*	Kruppel-like factor 4 (gut)	3	0
4094	*MAF*	v-maf musculoaponeurotic fibrosarcoma oncogene homolog (avian)	3	0
64651	*CSRNP1*	cysteine-serine-rich nuclear protein 1	1	0
2353	*FOS*	FBJ murine osteosarcoma viral oncogene homolog	1	0
1316	*KLF6*	Kruppel-like factor 6	1	0
467	*ATF3*	activating transcription factor 3	1	0
2114	*ETS2*	v-ets erythroblastosis virus E26 oncogene homolog 2 (avian)	1	0
6722	*SRF*	serum response factor (c-fos serum response element-binding transcription factor)	1	0
1999	*ELF3*	E74-like factor 3 (ets domain transcription factor, epithelial-specific)	1	0

Table S6. Enrichment analysis of Gene Ontology biological processes for genes regulated by key transcription factors identified by network analysis

GO BP	Term	P-value	Adjusted P-value	Count	Size
**HIF1A Upregulated genes**					
GO:0006986	response to unfolded protein	2,59E-15	4,09E-11	21	183
GO:0080135	regulation of cellular response to stress	2,10E-13	1,06E-09	37	778
GO:0006457	protein folding	2,97E-13	1,06E-09	21	232
GO:0010942	positive regulation of cell death	3,34E-13	1,06E-09	36	749
GO:0030155	regulation of cell adhesion	1,07E-12	1,87E-09	35	738
GO:0030335	positive regulation of cell migration	1,73E-11	2,48E-08	29	567
GO:0097190	apoptotic signaling pathway	2,17E-11	2,86E-08	30	612
GO:0097435	supramolecular fiber organization	5,43E-11	6,13E-08	32	719
GO:0002274	myeloid leukocyte activation	1,57E-10	1,38E-07	30	664
GO:1901987	regulation of cell cycle phase transition	4,05E-10	2,78E-07	25	486
GO:0001525	angiogenesis	2,18E-09	1,11E-06	27	611
GO:0030198	extracellular matrix organization	6,52E-09	2,88E-06	21	397
GO:0002253	activation of immune response	8,04E-09	3,34E-06	29	740
GO:0009611	response to wounding	1,21E-08	4,56E-06	28	708
GO:1903320	regulation of protein modification by small protein conjugation or removal	2,00E-08	6,31E-06	16	242
GO:0070201	regulation of establishment of protein localization	3,23E-08	9,60E-06	27	695
GO:0070848	response to growth factor	3,99E-08	1,12E-05	28	749
GO:0010035	response to inorganic substance	4,09E-08	1,13E-05	24	568
GO:0032496	response to lipopolysaccharide	6,06E-08	1,52E-05	18	334
GO:0008285	negative regulation of cell proliferation	1,14E-07	2,40E-05	28	788
**HIF1A Downregulated genes**					
GO:0016042	lipid catabolic process	9,74E-08	1,54E-03	13	343
GO:0006631	fatty acid metabolic process	3,20E-06	2,52E-02	12	396
GO:0000101	sulfur amino acid transport	6,31E-06	2,68E-02	3	7
GO:0014824	artery smooth muscle contraction	2,14E-05	4,13E-02	3	10
GO:0051705	multi-organism behavior	3,86E-05	4,13E-02	5	66
GO:1901605	alpha-amino acid metabolic process	4,81E-05	4,24E-02	8	225
GO:0008285	negative regulation of cell proliferation	4,95E-05	4,24E-02	15	788
**NKX2-3 Upregulated genes**					
GO:0007005	mitochondrion organization	2,49E-08	3,94E-04	33	560
GO:1903311	regulation of mRNA metabolic process	9,76E-08	7,71E-04	24	344
GO:0015936	coenzyme A metabolic process	2,36E-07	1,24E-03	6	15
GO:0016042	lipid catabolic process	3,64E-07	1,44E-03	23	343
GO:0002753	cytoplasmic pattern recognition receptor signaling pathway	9,19E-07	1,54E-03	10	69
GO:0006631	fatty acid metabolic process	1,25E-06	1,54E-03	24	396
GO:0006605	protein targeting	2,81E-06	2,55E-03	25	444
GO:0050688	regulation of defense response to virus	2,91E-06	2,55E-03	10	78
GO:0044257	cellular protein catabolic process	8,49E-06	4,97E-03	35	795
GO:0006914	autophagy	9,82E-06	5,35E-03	27	539
GO:0007041	lysosomal transport	1,19E-05	6,05E-03	11	111
GO:0034333	adherens junction assembly	1,58E-05	7,13E-03	10	94
GO:0009411	response to UV	2,27E-05	8,50E-03	12	141
GO:0010821	regulation of mitochondrion organization	3,31E-05	1,05E-02	14	195
GO:0071407	cellular response to organic cyclic compound	3,38E-05	1,05E-02	25	514
GO:0007031	peroxisome organization	3,59E-05	1,05E-02	9	83
GO:0070848	response to growth factor	3,69E-05	1,06E-02	32	749
GO:0061635	regulation of protein complex stability	4,30E-05	1,15E-02	4	11
GO:0030036	actin cytoskeleton organization	7,44E-05	1,68E-02	30	708
GO:0032870	cellular response to hormone stimulus	8,75E-05	1,89E-02	29	680
**ELF4 Upregulated genes**					
GO:0006914	autophagy	7,70E-12	6,08E-08	35	539
GO:0034330	cell junction organization	1,57E-10	8,29E-07	25	318
GO:0000422	autophagy of mitochondrion	5,74E-10	1,81E-06	13	79
GO:1903311	regulation of mRNA metabolic process	8,15E-10	1,84E-06	25	344
GO:0033674	positive regulation of kinase activity	1,09E-09	1,91E-06	34	615
GO:0042594	response to starvation	1,84E-09	2,53E-06	19	206
GO:0030036	actin cytoskeleton organization	3,21E-09	3,17E-06	36	708
GO:0007264	small GTPase mediated signal transduction	1,01E-08	6,97E-06	29	510
GO:0044257	cellular protein catabolic process	1,98E-08	1,30E-05	37	795
GO:0007163	establishment or maintenance of cell polarity	3,69E-08	2,33E-05	18	222
GO:0030155	regulation of cell adhesion	9,65E-08	4,26E-05	34	738
GO:1901137	carbohydrate derivative biosynthetic process	1,10E-07	4,47E-05	35	778
GO:0034976	response to endoplasmic reticulum stress	1,20E-07	4,47E-05	20	294
GO:0006644	phospholipid metabolic process	2,13E-07	6,74E-05	25	456
GO:0002274	myeloid leukocyte activation	2,70E-07	8,21E-05	31	664

GO:0031532	actin cytoskeleton reorganization	1,39E-06	3,27E-04	11	105
GO:0051223	regulation of protein transport	1,54E-06	3,58E-04	29	648
GO:0032386	regulation of intracellular transport	1,72E-06	3,88E-04	23	444
GO:0006935	chemotaxis	1,96E-06	4,32E-04	29	656
GO:0008360	regulation of cell shape	2,14E-06	4,45E-04	13	156

GO BP — *GO Biological process*
Count — *Real number of transcripts found associated with the GO term*
Size — *Population size of transcripts found associated wiht the GO term within the analysis*

Table S7. Oligonucleotide primer sequences used for quantitative PCR

Gene	Forward primer sequence	Reverse primer sequence
IRE1	5’-CGAAACTTCCTTTTACCATCCC-3’	5’-CGATGACAAAGTCTGCTGCTT-3’
HSPA1A/B	5’-AAGATCACCATCACCAACGA-3’	5’-TCCTCCGCTTTGTACTTCTC-3’
BIP	5'-CTGCTGTATCCTCTTCACCAGTTG- 3'	5'-TGACATTGAAGACTTCAAAGCTAAGA-3'
XBP1	5’-GGAGTTAAGACAGCGCTTGGGGA-3’	5’-TGTTCTGGAGGGGTGACAACTGGG-3’
XBP1s	5'-TGCTGAGTCCGCAGCAGGTG-3'	5'-GCTGGCAGGCTCTGGGGAAG-3'
CHOP	5'-GGAGCATCAGTCCCCCACTT-3'	5'-TGTGGGATTGAGGGTCACATC- 3'
GADD34	5'-CCCAGAAACCCCTACTCATGATC-3'	5'-GCCCAGACAGCCAGGAAAT-3'
ATF4	5'-CTCCGGGACAGATTGGATGTT-3'	5'-GGCTGCTTATTAGTCTCCTGGAC-3'
B2MG	5'-TCCATCCGACATTGAAGTTG-3'	5'-CGGCAGGCATACTCATCTT-3'
ACTB	5'-GCTGTGCTACGTCGCCCTG-3'	5'-GGAGGAGCTGGAAGCAGCC-3'
RPLP0	5’-GCAATGTTGCCAGTGTCTG-3’	5’-GCCTTGACCTTTTCAGCAA-3’
CYPA	5’-CTCGAATAAGTTTGACTTGTGTTT-3’	5’-CTAGGCATGGGAGGGAACA-3’
